# The association of body fat composition with risk of breast, endometrial, ovarian and colorectal cancers among normal weight participants in the UK Biobank

**DOI:** 10.1038/s41416-020-01210-y

**Published:** 2021-03-15

**Authors:** Rhonda S. Arthur, Andrew J. Dannenberg, Mimi Kim, Thomas E. Rohan

**Affiliations:** 1 Albert Einstein College of Medicine, Bronx, NY USA; 2 Weill Cornell Medical College, New York, NY USA

**Keywords:** Cancer, Risk factors

## Abstract

**Background:**

The association between body fat composition and risk of cancer in normal weight individuals (body mass index (BMI) 18.5–24.9 kg/m^2^) is unclear.

**Methods:**

We examined the association of measures of adiposity with risk of incident cancers of the breast (postmenopausal), endometrium, ovary and colon/rectum among 149,928 normal weight individuals (40–70 years) who were enrolled in the UK Biobank cohort between 2006 and 2010.

**Results:**

All of the body fat measures were positively associated with invasive postmenopausal breast cancer risk (hazard ratios (HR) for the uppermost quintile (Q5) versus the lowest quintile (Q1) ranged from 1.32 (95% CI: 1.09–1.60) for waist circumference (WC) to 1.56 (1.28–1.90) for BMI). Trunk fat mass index (HR_Q5 vs Q1_: 1.72, 95% CI: 1.02–2.89) and WC (HR_Q5 vs Q1_: 1.65, 95% CI: 1.01–2.70)) were positively associated with risk of endometrial cancer. Among males, trunk fat:trunk fat free mass ratio, trunk fat:leg fat mass ratio and (HR_Q5 vs Q1_: 1.63, 95% CI: 1.02–2.60; 1.92, 1.20–3.07 and 1.68, 1.05–2.66, respectively) were positively associated with colon cancer risk. None of the body fat measures was associated with risk of ovarian cancer or colorectal cancer in women.

**Conclusion:**

The findings of this study suggest that the current normal weight category based on BMI includes individuals who are at increased risk of some obesity-related cancers.

## Background

Adiposity is recognised as a risk factor for at least 13 different types of cancer.^1^ To determine body fat levels, most epidemiological studies utilise body mass index (BMI; calculated as weight (kg)/height (m^2^)) as it is a relatively inexpensive and easy to obtain measure.^2^ However, BMI does not provide precise information on the extent and distribution of body fatness,^2,3^ which are key determinants of cancer risk. Hence, estimates of cancer risk associated with BMI may be subject to misclassification.

Excess adiposity, particularly centrally, is purported to induce inflammation and metabolic dysfunctions which can promote carcinogenesis.^4^ Interestingly, an increasing number of epidemiological studies has suggested that excess body fat and metabolic dysfunction may contribute to increased risk of some obesity-related cancers among individuals who are classified as normal weight (i.e. BMI 18.5–24.9 kg/m^2^). In a prospective study in the Women’s Health Initiative cohort and an earlier Canadian case-control study, general and/or central adiposity were positively associated with risk of postmenopausal breast cancer^5^ and endometrial cancer^6^ among normal weight women. Moreover, in the few epidemiological studies, which have examined the associations between various metabolic dysfunctions and cancer among normal weight individuals, those with hyperinsulinemia^7^ and/or metabolic syndrome, common sequelae of excess body fat, were observed to have increased risk of breast cancer,^7,8^ colorectal cancer^9^ and endometrial cancer.^6^ Together, these studies provide evidence to suggest that despite being classified as normal weight according to BMI, individuals with excess body fat (generally or regionally) and the resulting metabolic disturbances may have increased cancer risk.^5–8^

In addition to BMI, which is utilised as a proxy for overall body fat, dual energy X-ray absorptiometry (DXA),^10^ magnetic resonance imaging (MRI), computer tomography (CT) and bioelectrical impedance analysis (BIA) are used, not only to evaluate overall body fat, but also to analyse body fat composition centrally and peripherally. MRI, CT and, to a lesser extent, DXA are among the most robust methods for assessing body fat composition.^10^ However, these methods are not commonly used in epidemiological settings due to their high cost. BIA, a less robust method for measuring body fat composition than the aforementioned methods, is a relatively simple, non-invasive, inexpensive technique.^11^ This procedure not only assesses overall body fat, but also yields measures of body fat percent as well as levels of body fat in the central and peripheral regions.^11^ Given the putative importance of body fat in influencing cancer risk, we used data from the UK Biobank, a large prospective study in which BIA measures of body fat composition were obtained for virtually all participants, to examine the association of measures of overall, central, and peripheral adiposity with risk of four of the most commonly diagnosed obesity-related cancers, namely, postmenopausal breast cancer, endometrial cancer, ovarian cancer and colorectal cancer, among normal weight individuals.

## Methods

### Study population and design

The UK Biobank cohort comprises 503,317 participants, aged 40–69 at enrolment, who were recruited between 2006 and 2010 from across 22 centres located throughout England, Wales, and Scotland (http://www.ukbiobank.ac.uk).^12–14^ The study was approved by the North West Multi-center Research Ethics Committee, the National Information Governance Board for Health and Social Care in England and Wales, and the Community Health Index Advisory Group in Scotland. All participants provided written informed consent.

At enrolment, a self-administered touchscreen questionnaire and nurse-led interview were used to collect information on the participants’ socio-demographic characteristics, health and medical history, reproductive factors, and diet and lifestyle (http://www.ukbiobank.ac.uk/resources/). Further details on the ascertainment of the aforementioned variables are presented in the eMethods (See Supplementary Material).

### Body composition and anthropometric measures

At the enrolment visit, the Tanita BC418MA body composition analyser (Tanita, Tokyo, Japan) was used to measure the participants’ whole body fat mass, whole body fat percentage, whole body fat free mass, trunk fat mass, trunk fat percentage, trunk fat free mass and leg fat mass. The participants’ waist and hip circumferences were measured using Wessex nonstretchable sprung tape measures (Wessex, United Kingdom), and standing height was determined using the Seca 202 device (Seca, Hamburg, Germany). BMI, body fat mass index (FMI;^15,16^) and trunk fat mass index (TFMI^15,16^) were calculated by dividing weight (kg), whole body fat mass (kg.) and trunk fat mass (kg.), respectively, by the square of standing height (m^2^). Waist to hip ratio (WHR) was calculated by dividing waist circumference by the corresponding hip circumference.

### Outcome ascertainment

The endpoints of interest in this study were incident breast (postmenopausal: International Classification of Diseases (ICD)-10 code: C50), endometrial (ICD 10 code: C54.1), ovarian (ICD 10 code: C56) and colorectal (ICD 10 code: C18-C20) cancers. In the UK Biobank, cancer diagnoses are ascertained through linkage to national cancer registries in England, Wales and Scotland. For the present study, complete follow-up was available through 31 March 2016 for England and Wales and 31 October 2015 for Scotland.

### Analytical cohort

Participants were excluded from the study if they (1) withdrew consent (*N* = 811); (2) did not have a BMI in the normal range (i.e. not between 18.5 kg/m^2^ and 24.9 kg/m^2^; *N* = 342,668); (3) did not have BIA measures (*N* = 2176) or (4) had a history of cancer except for nonmelanoma skin cancer (N = 7734). As hormone replacement therapy (HRT) use is known to attenuate the associations of adiposity with risk of female reproductive cancers (i.e. breast, endometrial and ovarian cancers),^17,18^ we excluded from analyses of these cancers current HRT users or those whose HRT status was unknown (*N* = 9450). For breast cancer, women who were premenopausal or had unknown menopausal status were also excluded (*N* = 35,284), leaving a total of 1051 cases and 51,678 noncases. For endometrial cancer, we also excluded women with a history of hysterectomy or those with unknown hysterectomy status (*N* = 10,275), leaving a total of 155 cases and 77,583 noncases, while for ovarian cancer, we also excluded women with a history of bilateral oophorectomy or those with unknown oophorectomy status (*N* = 4631), leaving a total of 170 cases and 83,212 noncases. After exclusions, for colorectal cancer, a total of 843 cases and 149,085 noncases remained for analysis.

### Statistical analyses

Cox proportional hazards regression models were used to estimate hazard ratios (HRs) and 95% confidence intervals (CI) for the associations of the body fat measures with risk of the cancers of interest. The body fat measures were analysed following categorisation by quintiles (sex-specific quintiles for the body fat measures were based on the distribution of the body fat measures in the noncases). To make comparisons between the HRs for the associations between the body fat measures and the cancers of interest, we also estimated the HRs per standard deviation (SD) increase in the body fat measures. Time to diagnosis of the selected cancers was the underlying timescale. Participants were followed up from their date of enrolment until the date of cancer diagnosis (except for non‐melanoma skin cancer), date of withdrawal from the study, date of death, or until the end of follow-up, whichever came first (participants were censored if they did not develop the end-point of interest by the end of follow-up, died, or withdrew from the study before the end of follow-up). All models were adjusted for age at recruitment (years; continuous), socioeconomic status (based on Townsend deprivation index categorised by quintiles^19^), ethnicity (whites, other, missing), alcohol consumption (never, special occasions/1–3 times weekly, 3–4 times weekly, daily/almost daily), height (cm; continuous); physical activity (MET-min/week; continuous), sex (except for analyses of the sex-specific cancers; male/female) and smoking status (never, former, current, missing). For female reproductive cancers, the models were additionally adjusted for age at menarche (years; <12, 12–13, 14+, missing), parity and age (years) at first live birth combined (nulliparous, ≥25 and <3 live births, ≥25 and ≥3 live births, <25 and <3 live births, <25 and ≥3 live births, missing), HRT status (never/former), age (years) at menopause (years; premenopausal, ≤45, 46–50, 51–54, ≥55, missing), family history of breast cancer (yes, no, missing (breast cancer only)) and mammogram screening (yes, no, missing; breast cancer only). For colorectal cancer, models were additionally adjusted for sex (male, female), family history of bowel cancer (yes, no, missing), history of diabetes (yes, no, missing), red meat intake (none, <once/week, 1–<2 times/week, 2–<5 times/week, ≥5 times/week), processed meat intake (none, <once/week, once/week, 2–4 times/week, ≥5 times/week), fruits and vegetables intake (<1 serving/day, 1–<3 servings/day, 3–<4 servings/day, 4–<5 servings/day, ≥5 servings/day), vitamin D supplement use (yes, no, missing), folate supplement use (yes, no, missing), and use of ibuprofen (yes, no) and aspirin (yes, no). A missing value indicator was included for variables with missing values (See number of missing values in the footnote to Table 1). In separate models, we additionally adjusted the BIA-derived variables for BMI to assess the effect of BMI on the association between BIA-derived body fat measures and risk of the cancers of interest. We also assessed the associations of WC and WHR (using standard categorisations^20,21^) with risk of the cancers of interest (For men, the two highest categories were collapsed due to the small number of cases with high WC or WHR).

**Table 1:** Characteristics of participants in the UK Biobank.

Characteristics	All				Females				Males			
	FMI		TFMI		FMI		TFMI		FMI		TFMI	
	Q1	Q5	Q1	Q5	Q1	Q5	Q1	Q5	Q1	Q5	Q1	Q5
Age at enrollment (yrs; Median (IQR))	53 (46–60)	60 (53–64)	53 (46–60)	60 (53–64)	53 (46–60)	59 (53–64)	53 (46–60)	59 (52–64)	54 (47–61)	61 (53–65)	54 (46–61)	61 (55–65)
Socioeconomic status (lowest quintile; %)	17.1	18.4	17.1	18.6	17.7	19.3	17.7	19.5	15.9	16.7	15.9	17.0
Family history of breast cancer (%)	–	–	–	–	10.4	11.2	10.9	11.5	–	–	–	–
Age at menarche (<12 yrs.; %)	–	–	–	–	13.2	14.5	13.9	13.8	–	–	–	–
Nulliparous: (%)	–	–	–	–	24.6	18.1	23.6	19.2	–	–	–	–
Age at menopause (>55 yrs.; %)	–	–	–	–	10.4	12.9	10.6	12.6	–	–	–	–
HRT use ever: (never; %)	–	–	–	–	64.0	54.6	63.9	55.1	–	–	–	–
Mammogram ever (%)	–	–	–	–	94.0	96.1	93.5	95.7	–	–	–	–
Alcohol ((≥5 times/week; %)	22.3	22.4	21.9	22.7	20.8	19.0	20.4	19.2	24.9	28.7	24.7	29.4
Physical activity (MET-min/week; Median (IQR))	37.8 (19.1–71.1)	28.4 (13.6–55.8)	38.6 (19.4–72.4)	27.8 (13.5–54.4)	37.0 (18.5–67.9)	28.2 (13.6–55.1)	37.1 (18.5–69.1)	28.0 (13.6–54.2)	39.6 (19.9–76.9)	28.8 (13.6–57.4)	41.2–21.0–80.5)	27.6 (12.9–54.7)
BMI (kg/m^2^; Median (IQR))	21.1 (20.2–22.1)	23.9 (23.6–24.7)	21.3 (20.2–22.4)	24.2 (23.7–24.6)	20.7 (19.9–21.6)	24.3 (23.9–24.7)	20.9 (19.9–21.9)	24.2 (23.6–24.6)	22.0 (20.9–23.0)	24.3 (23.8–24.7)	22.1 (20.9–23.2)	24.3 (23.7–24.7)
WC (cm.; Median (IQR))	72 (68–79)	83 (78–89)	72 (68–79)	83 (78–89)	69 (66–72)	80 (76–83)	69 (67–73)	80 (76–83)	81 (77–85)	90 (86–94)	81 (77–85)	90 (87–94)
WHR (median (IQR))	0.79 (0.74–0.84)	0.84 (0.78–0.90)	0.79 (0.74–0.84)	0.84 (0.78–0.90)	0.76 (0.73–0.79)	0.80 (0.76–0.84)	0.76 (0.73–0.79)	0.80 (0.76–0.88)	0.85 (0.82–0.89)	0.92 (0.88–0.95)	0.85 (0.82–0.89)	0.92 (0.89–0.95)
Smoking (never; %)	60.8	56.5	60.4	56.6	62.6	59.9	62.5	60.1	57.4	50.1	56.5	49.9
Diabetes (%)	1.6	2.6	1.7	2.6	1.3	1.4	1.4	1.4	2.3	4.8	2.3	5.0
Aspirin use ever (%)	6.6	10.9	6.6	10.9	5.2	7.8	5.3	7.4	9.2	16.7	9.2	17.5
Ibuprofen use ever (%)	11.1	11.1	11.3	10.9	12.5	12.8	12.7	12.7	8.5	7.9	8.6	7.6
Vitamin D use (%)	5.3	4.9	5.3	4.9	6.6	5.8	6.5	5.7	3.1	3.2	3.1	3.4
Folate use ever (%)	2.5	2.4	2.5	2.5	3.0	2.7	3.0	2.9	1.6	1.7	1.5	1.8
Fruits and vegetable intake (≥5 servings/dy; %)	28.5	25.6	28.8	25.7	31.4	28.9	31.6	29.1	23.1	19.5	23.5	19.2
Red meat (≥5 servings/dy; %)	3.8	4.3	3.8	4.1	3.0	3.5	3.1	3.3	5.2	5.9	5.2	5.6
Processed meat (≥5 servings/dy; %)	3.7	3.0	3.7	3.0	1.7	1.6	1.7	1.6	7.3	5.7	7.4	5.6

Number missing: ethnicity (*n* = 623); smoking status (*n* = 558); age at menarche (*n* = 3099); parity and age at first live birth combined (*n* = 13,265); age at menopause (*n* = 7515); family history of breast cancer (*n* = 5257); family history of bowel cancer (*n* = 16,464); history of diabetes (*n* = 313); vitamin D supplement use (*n* = 564); folate supplement use (*n* = 564).

*FMI* fat mass index, *TFMI* trunk fat mass index, *HRT* hormone replacement therapy, *MET* metabolic equivalent, *BMI* body mass index, *IQR* interquartile range, *WC* waist circumference, *WHR* waist to hip ratio.

Excess adiposity, risk of endometrial cancer^1,22^ and risk of ovarian cancer^23^ increase with age. Therefore, we also examined the associations of the body fat measures with risk of the aforementioned cancers among postmenopausal women (the number of cases among premenopausal women were too small for us to perform any meaningful analyses in this stratum). Further, there is some evidence that the association between body fat and risk of colorectal cancer varies by sex.^24^ Hence, we also conducted the analyses separately for men and women. We included an interaction term in the regression models and tested its coefficient using the Wald test in order to determine whether sex is an effect modifier of the associations between the body fat measures and risk of colorectal cancer.

The proportional hazards assumption was not violated as evidenced by testing using Schoenfeld residuals. *P* values for trend (P-trend) were estimated by including the ordinal variables for the body fat measures as continuous variables in the regression models and testing their coefficients using Wald tests.

To address the possibility of reverse causation, we conducted sensitivity analyses in which we excluded participants with a follow-up time of two years or less.

All statistical analyses were performed using Stata 16.1 (StataCorp, College Station, TX, USA). All *P* values were two-sided.

## Results

After a median follow-up time of 7 years (interquartile range: 6.4–7.7 years), 1051, 155, 170 and 843 incident postmenopausal breast, endometrial, ovarian and colorectal cancer cases had been ascertained. In the overall analytical cohort, participants with FMI and TFMI in the uppermost quintiles had higher BMI, WC, WHR and red meat intake than those in the lowest quintiles but had lower physical activity levels and fruit and vegetable intake (Table 1). Similar results were seen in sex-specific analyses (Table 1).

In males, BIA-derived measures had mostly moderate correlations with BMI. Among females, most of the BIA-derived measures of general and central adiposity had moderate to strong correlations with BMI (Table 2).

**Table 2:** Spearman correlation (R) between BIA-derived and anthropometric measures of adiposity among participants in the UK Biobank.

	FMI	Body fat%	Whole body fat to whole body fat free mass	BMI	TFMI	Trunk fat %	Trunk fat to trunk fat free mass	Leg fat	Trunk to leg	WC	WHR
Men											
FMI	1.00										
Body fat%	0.94	1.00									
Whole body fat to whole body fat free mass	0.98	0.96	1.00								
BMI	0.58	0.45	0.42	1.00							
TFMI	0.95	0.99	0.95	0.52	1.00						
Trunk fat %	0.92	0.99	0.95	0.39	0.98	1.00					
Trunk fat to trunk fat free mass	0.92	0.99	0.95	0.39	0.98	0.99	1.00				
Leg fat	−0.14	−0.23	−0.24	0.33	−0.16	−0.22	−0.22	1.00			
Trunk to leg	0.91	0.99	0.95	0.38	0.98	0.98	0.98	−0.27	1.00		
WC	0.54	0.51	0.48	0.53	0.54	0.48	0.48	0.25	0.50	1.00	
WHR	0.43	0.44	0.42	0.31	0.44	0.42	0.42	−0.11	0.43	0.75	1.00
Women											
FMI	1.00										
Body fat%	0.96	1.00									
Whole body fat to whole body fat free mass	0.96	0.99	1.00								
BMI	0.81	0.64	0.64	1.00							
TFMI	0.97	0.97	0.97	0.70	1.00						
Trunk fat %	0.91	0.97	0.97	0.56	0.98	1.00					
Trunk fat to trunk fat free mass	0.91	0.96	0.97	0.55	0.98	0.99	1.00				
Leg fat	0.07	−0.03	−0.03	0.28	0.16	0.10	0.10	1.00			
Trunk to leg	0.92	0.98	0.98	0.56	0.97	0.98	0.98	0.04	1.00		
WC	0.61	0.56	0.56	0.56	0.58	0.53	0.52	0.20	0.55	1.00	
WHR	0.28	0.25	0.25	0.24	0.23	0.20	0.21	−0.10	0.23	0.79	1.00

*BMI* body mass index, *FMI* fat mass index, *TFMI* trunk fat mass index, *WC* waist circumference, *WHR* waist to hip ratio.

As shown in Table 3, all BIA-derived body fat measures were positively associated with risk of invasive breast cancer among normal weight postmenopausal women. Compared to those in the lowest quintiles (Q1), those with BIA-derived measures of overall adiposity in the highest quintiles (Q5) had 34% to 46% increases in risk (HRs ranging from 1.34 (95% CI: 1.09–1.64) for body fat% to 1.46 (95% CI: 1.20–1.78) for FMI). With respect to the BIA-derived measures of central and peripheral adiposity, the increases in risk of breast cancer ranged from 26% to 47% (HR ranging from 1.42 (95% CI: 1.16–1.75) for trunk fat% to 1.47 (95% CI: 1.20–1.80) for TFMI) (Table 3). A relatively high BMI (HR_Q5 vs Q1_: 1.56, 95% CI: 1.28–1.90) and a relatively high WC (HR_Q5 vs Q1_: 1.32, 95% CI: 1.09–1.60) were also positively associated with risk of breast cancer in postmenopausal women (Table 3). For endometrial cancer, the highest quintile levels of TFMI and WC were positively associated with risk (HR_Q5 vs Q1_: 1.72; 95% CI: 1.02–2.89 and HR_Q5 vs Q1_: 1.65; 95% CI: 1.01–2.70, respectively), but the trends in risk across quintile levels were not statistically significant (Table 3). In analyses restricted to postmenopausal women, those with TFMI and WC in the highest quintile had a twofold and 93% increases in risk of endometrial cancer, respectively (HR_Q5 vs Q1_: 2.30; 95% CI: 1.27–4.15 and HR_Q5 vs Q1_: 1.93; 95% CI: 1.09–3.41, respectively; Supplementary Table 1). When considering the standard categorisations for WC and WHR, high WC (>88 cm), but not WHR (≥0.85), was positively associated with risk of postmenopausal breast cancer. With respect to endometrial cancer (overall), high WHR, but not high WC, was positively associated with risk (Supplementary Table 2). However, when we restricted the analyses to postmenopausal women, both high WC (≥88 cm) and high WHR (≥0.85) were associated with approximately twofold increases in risk of endometrial cancer (Supplementary Table 2). None of the body fat measures was associated with ovarian cancer risk among normal weight women (Table 3, Supplementary Tables 1 and 2).

**Table 3:** Hazard ratios and 95% CI for the association of BIA-derived baseline body fat measures with risk of incident, invasive female-specific cancers among women in the UK Biobank.

	Breast (postmenopausal)^a^ *N* = 1051		Endometrium *N* = 155		Ovary *N* = 170	
	Cases/incidence per 1000PY	Multivariable-adjusted HR (95% CI)	Cases/incidence per 1000PY	Multivariable-adjusted HR (95% CI)	Cases/incidence per 1000PY	Multivariable-adjusted HR (95% CI)
FMI (kg/m^2^)						
Q1	165/2.25	1.00	31/0.23	1.00	35/0.25	1.00
Q2	182/2.50	1.09 (0.88–1.35)	31/0.26	1.08 (0.66–1.78)	43/0.34	1.32 (0.84–2.06)
Q3	207/2.89	1.25 (1.01–1.54)	26/0.25	0.98 (0.58–1.65)	25/0.22	0.81 (0.48–1.35)
Q4	235/3.14	1.34 (1.10–1.64)	34/0.34	1.27 (0.78–2.08)	40/0.37	1.28 (0.81–2.02)
Q5	262/3.53	1.46 (1.20–1.78)	33/0.39	1.32 (0.80–2.18)	27/0.29	0.90 (0.54–1.51)
P_trend_^b^		<0.001		0.21		0.73
Per SD increase		1.15 (1.08–1.22)		1.14 (0.96–1.34)		0.99 (0.85–1.16)
Body fat %						
Q1	157/2.28	1.00	29/0.22	1.00	38/0.27	1.00
Q2	184/2.48	1.06 (0.86–1.31)	35/0.29	1.21 (0.74–1.99)	35/0.27	0.92 (0.58–1.45)
Q3	226/3.01	1.27 (1.04–1.56)	26/0.24	0.96 (0.56–1.65)	29/0.25	0.80 (0.49–1.31)
Q4	228/3.15	1.29 (1.05–1.59)	29/0.31	1.17 (0.69–1.97)	41/0.40	1.21 (0.77–1.90)
Q5	256/3.36	1.34 (1.09–1.64)	36/0.42	1.45 (0.87–2.41)	27/0.28	0.78 (0.47–1.30)
P_trend_^b^		0.001		0.23		0.78
Per SD increase		1.13 (1.06–1.21)		1.13 (0.95–1.34)		0.99 (0.85–1.16)
Ratio of whole body fat mass to whole body fat free mass						
Q1	154/2.26	1.00	29/0.22	1.00	37/0.27	1.00
Q2	186/2.51	1.08 (0.88–1.34)	35/0.29	1.21 (0.73–1.97)	35/0.27	0.93 (0.59–1.48)
Q3	221/2.98	1.27 (1.03–1.57)	24/0.22	0.89 (0.52–1.54)	28/0.24	0.79 (0.48–1.30)
Q4	223/3.14	1.30 (1.06–1.60)	31/0.34	1.26 (0.75–2.11)	43/0.43	1.31 (0.84–2.06)
Q5	267/3.38	1.36 (1.11–1.66)	36/0.41	1.37 (0.83–2.30)	27/0.27	0.77 (0.46–1.28)
P_trend_^b^		0.001		0.24		0.85
Per SD increase		1.12 (1.05–1.20)		1.12 (0.95–1.32)		0.99 (0.84–1.16)
BMI (kg/m^2^)						
Q1	173/2.22	1.00	33/0.26	1.00	39/0.29	1.00
Q2	208/2.83	1.27 (1.04–1.55)	26/0.23	0.86 (0.51–1.44)	31/0.26	0.86 (0.54–1.38)
Q3	209/2.86	1.30 (1.06–1.59)	34/0.32	1.17 (0.73–1.90)	36/0.31	1.01 (0.65–1.61)
Q4	216/3.05	1.38 (1.13–1.69)	25/0.25	0.91 (0.54–1.53)	33/0.31	0.97 (0.61–1.55)
Q5	245/3.43	1.56 (1.28–1.90)	37/0.39	1.36 (0.85–2.195)	31/0.30	0.92 (0.57–1.48)
P_trend_^b^		<0.001		0.22		0.91
Per SD increase		1.16 (1.08–1.23)		1.13 (0.96–1.33)		1.00 (0.86–1.17)
TFMI (kg/m^2^)						
Q1	163/2.25	1.00	27/0.20	1.00	40/0.29	1.00
Q2	190/2.50	1.07 (0.87–1.32)	36/0.30	1.36 (0.82–2.25)	33/0.25	0.87 (0.55–1.37)
Q3	208/2.83	1.19 (0.97–1.46)	26/0.25	1.07 (0.62–1.85)	31/0.29	0.80 (0.49–1.49)
Q4	222/3.06	1.25 (1.02–1.54)	28/0.29	1.21 (0.70–2.08)	42/0.37	1.11 (0.71–1.76)
Q5	268/3.71	1.47 (1.20–1.80)	38/0.45	1.72 (1.02–2.89)	24/0.27	0.84 (0.50–1.39)
P_trend_^b^		<0.001		0.11		0.88
Per SD increase		1.16 (1.08–1.23)		1.16 (0.98–1.37)		0.99 (0.84–1.16)
Trunk fat %						
Q1	158/2.25	1.00	32/0.24	1.00	39/0.28	1.00
Q2	202/2.51	1.07 (0.87–1.32)	31/0.24	0.92 (0.56–1.51)	28/0.21	0.68 (0.42–1.10)
Q3	211/2.95	1.23 (0.99–1.52)	24/0.23	0.82 (0.48–1.40)	38/0.34	1.07 (0.68–1.69)
Q4	216/3.00	1.21 (0.98–1.50)	30/0.32	1.09 (0.65–1.83)	40/0.39	1.18 (0.75–1.87)
Q5	264/3.66	1.42 (1.16–1.75)	38/0.45	1.40 (0.84–2.32)	25/0.27	0.75 (0.44–1.28)
P_trend_^b^		<0.001		0.14		0.91
Per SD increase		1.13 (1.05–1.21)		1.16 (0.97–1.38)		0.98 (0.84–1.16)
Ratio of trunk fat mass to trunk fat free mass						
Q1	156/2.28	1.00	31/0.24	1.00	40/0.28	1.00
Q2	195/2.44	1.03 (0.83–1.27)	31/0.24	0.92 (0.56–1.52)	34/0.26	0.64 (0.39–1.05)
Q3	218/2.99	1.23 (0.99–1.51)	24/0.23	0.82 (0.48–1.40)	29/0.25	1.03 (0.65–1.62)
Q4	211/2.97	1.19 (0.96–1.47)	30/0.32	1.11 (0.66–1.87)	39/0.37	1.16 (0.73–1.84)
Q5	271/3.65	1.41 (1.14–1.73)	39/0.45	1.42 (0.85–2.36)	28/0.30	0.74 (0.44–1.25)
P_trend_^b^		<0.001		0.11		0.93
Per SD increase		1.12 (1.05–1.19)		1.14 (0.97–1.34)		0.98 (0.83–1.15)
Leg fat mass (kg)						
Q1	179/2.24	1.00	34/0.24	1.00	41/0.33	1.00
Q2	205/2.64	1.17 (0.95–1.43)	31/0.26	1.01 (0.62–1.65)	36/0.32	0.93 (0.60–1.46)
Q3	215/2.81	1.22 (1.00–1.49)	31/0.28	1.05 (0.64–1.71)	34/0.29	0.95 (0.60–1.49)
Q4	233/3.31	1.40 (1.15–1.70)	30/0.32	1.15 (0.70–1.90)	36/0.30	0.79 (0.48–1.30)
Q5	219/3.50	1.37 (1.12–1.69)	29/0.23	1.26 (0.74–2.14)	23/0.21	1.12 (0.69–1.85)
P_trend_^b^		<0.001		0.33		0.99
Per SD increase		1.14 (1.07–1.22)		1.10 (0.93–1.31)		1.01 (0.86–1.18)
Ratio of trunk fat mass to leg fat mass						
Q1	174/2.32	1.00	29/0.23	1.00	40/0.30	1.00
Q2	214/2.74	1.12 (0.92–1.38)	35/0.29	1.21 (0.73–1.99)	25/0.18	0.77 (0.47–1.27)
Q3	207/2.76	1.09 (0.88–1.35)	21/0.20	0.77 (0.43–1.38)	37/0.33	1.12 (0.70–1.81)
Q4	196/2.67	1.02 (0.82–1.28)	33/0.32	1.21 (0.70–2.09)	37/0.37	1.13 (0.68–1.88)
Q5	260/3.98	1.45 (1.15–1.83)	37/0.41	1.50 (0.83–2.70)	31/0.33	1.10 (0.62–1.95)
P_trend_^b^		0.01		0.25		0.39
Per SD increase		1.11 (1.03–1.19)		1.11 (1.004–1.22)		0.98 (0.81–1.18)
WC (cm)						
Q1	203/2.39	1.00	32/0.22	1.00	44/0.29	1.00
Q2	226/2.55	1.04 (0.86–1.25)	47/0.34	1.48 (0.95–2.33)	34/0.23	0.77 (0.49–1.21)
Q3	205/3.03	1.20 (0.99–1.46)	20/0.20	0.86 (0.49–1.51)	39/0.37	1.19 (0.77–1.84)
Q4	165/3.09	1.20 (0.98–1.48)	19/0.26	1.08 (0.61–1.93)	23/0.29	0.92 (0.55–1.53)
Q5	252/3.50	1.32 (1.09–1.60)	37/0.42	1.65 (1.01–2.70)	30/0.31	0.91 (0.56–1.47)
P_trend_^b^		0.001		0.23		0.99
Per SD increase		1.13 (1.07–1.21)		1.17 (0.99–1.38)		0.98 (0.84–1.15)
WHR						
Q1	186/2.57	1.00	35/0.28	1.00	33/0.25	1.00
Q2	197/2.76	1.08 (0.88–1.32)	27/0.24	0.84 (0.51–1.38)	46/0.38	1.47 (0.95–2.32)
Q3	212/2.97	1.15 (0.94–1.40)	36/0.34	1.20 (0.76–1.91)	25/0.22	0.83 (0.49–1.39)
Q4	216/2.88	1.12 (0.92–1.36)	23/0.23	0.77 (0.46–1.31)	41/0.37	1.31 (0.82–2.09)
Q5	240/3.14	1.21 (0.99–1.47)	33/0.35	1.19 (0.73–1.92)	25/0.24	0.80 (0.47–1.36)
P_trend_^b^		0.06		0.63		0.37
Per SD increase		1.06 (1.01–1.13)		1.14 (0.98–1.34)		0.80–1.09)

All models were adjusted for age at enrollment, education, age at menarche, age at first full-term birth and parity combined, HRT status, age at menopause, height, physical activity, alcohol intake, smoking.

Ranges:

FMI: ≤ 6.0, 6.1–6.9, 7.0–7.6, 7.7–8.4, >8.4; body fat %: ≤27.4, 27.5–30.5, 30.6–32.8, 32.9–35.1, >35.1; whole body fat to whole body fat free mass: ≤0.38, 0.39–0.44, 0.45–0.49, 0.50–0.54, >0.54; BMI: ≤ 21.4, 21.5–22.6, 22.7–23.5, 23.6–24.2, >24.2; TFMI: ≤ 2.86; 2.87–3.44, 3.45–3.90, 3.91–4.39, >4.39; trunk fat %: ≤23.8, 23.9–27.8, 27.9–30.6, 30.7–33.6, >33.6; trunk fat mass to trunk fat free mass: ≤0.31, 0.32–0.38, 0.39–0.44, 0.45–0.50, >0.50; leg fat mass: ≤6.8, 6.9–7.6, 7.7–8.2, 8.3–8.8, >8.8; ratio of trunk fat mass to leg fat mass: ≤1.06, 1.07–1.19, 1.20–1.30, 1.31–1.42, >1.43; WC: ≤ 70, 71–74, 75–77, 78–80.3, >80.3; WHR: ≤ 0.74, 0.75–0.77, 0.78–0.80, 0.81–0.83, >0.83 for quintiles 1,2 3, 4 and 5, respectively.

*PY* person-year, *CI* confidence interval, *HR* hazard ratio, *SD* standard deviation, *FMI* fat mass index, *TFMI* trunk fat mass index.

^a^Also adjusted for family history of breast cancer and mammogram ever.

^b^All tests were two-sided.

The associations of the BIA-derived body fat measures with risk of colorectal cancer among normal weight participants are shown in Table 4 and Supplementary Tables 2–4. In analyses based on quintiles, none of the body fat measures was associated with risk of colorectal, colon or rectal cancer among men and women combined (Table 4). However, among men, ratio of trunk fat mass to leg fat mass and WHR in the highest quintile were associated with increased risk of colorectal cancer among men (HR_Q5 vs Q1_: 1.63; 95% CI: 1.14–2.32 and HR_Q5 vs Q1_: 1.40; 95% CI: 1.01–1.94, respectively). In analyses restricted to colon cancer, the ratio of trunk fat to trunk fat free mass (HR_Q5 vs Q1_: 1.63; 95% CI: 1.02–2.60), the ratio of trunk fat mass to leg fat mass (HR_Q5 vs Q1_: 1.93; 95% CI: 1.01–1.33), and WC (HR_Q5 vs Q1_: 1.68; 95% CI: 1.05–2.66) were positively associated with risk of colon cancer among men (Supplementary Table 3). Our analyses using a standard categorisation also showed that moderate to high WC (≥90 cm) was positively associated with risk of colorectal cancer among men. There were no associations between the body fat measures and risk of colorectal cancer among women (Supplementary Tables 2 and 4). Our formal test for heterogeneity indicated that the associations of trunk fat to trunk fat free mass (p for heterogeneity = 0.026) and the ratio of trunk fat mass to leg fat mass (p for heterogeneity = 0.014) with risk of colon cancer differed by sex (Supplementary Table 4).

**Table 4:** Hazard ratios and 95% CI for the association of baseline BIA-derived baseline body fat measures with risk of incident, invasive colorectal cancer among men and women (combined) in the UK Biobank.

Body fat measures	Colorectal *N* = 843		Colon *N* = 541		Rectal *N* = 302	
	Cases/incidence per 1000PY	Multivariable-adjusted HR (95% CI)	Cases/incidence per 1000PY	Multivariable-adjusted HR (95% CI)	Cases/incidence per 1000PY	Multivariable-adjusted HR (95% CI)
FMI (kg/m^2^)						
Q1	161/0.64	1.00	104/0.41	1.00	57/0.23	1.00
Q2	161/0.73	1.05 (0.84–1.31)	104/0.47	1.04 (0.80–1.37)	57/0.26	1.06 (0.73–1.53)
Q3	178/0.87	1.16 (0.94–1.44)	116/0.56	1.17 (0.90–1.53)	62/0.30	1.15 (0.80–1.66)
Q4	169/0.89	1.13 (0.91–1.40)	106/0.56	1.08 (0.82–1.42)	63/0.33	1.22 (0.85–1.75)
Q5	174/1.00	1.14 (0.92–1.42)	111/0.64	1.11 (0.85–1.46)	63/0.36	1.19 (0.83–1.72)
P_trend_^a^		0.18		0.44		0.24
Body fat %						
Q1	155/0.63	1.00	98/0.40	1.00	57/0.23	1.00
Q2	160/0.70	0.99 (0.80–1.25)	109/0.48	1.07 (0.81–1.41)	51/0.22	0.87 (0.60–1.28)
Q3	188/0.90	1.19 (0.96–1.48)	116/0.55	1.15 (0.88–1.51)	72/0.34	1.27 (0.89–1.80)
Q4	154/0.84	1.02 (0.82–1.28)	102/0.55	1.06 (0.80–1.40)	52/0.28	0.96 (0.66–1.41)
Q5	186/1.07	1.17 (0.94–1.45)	116/0.66	1.14 (0.86–1.50)	70/0.40	1.23 (0.86–1.76)
P_trend_^a^		0.18		0.44		0.22
Whole body fat mass to whole body fat free mass						
Q1	154/0.64	1.00	98/0.41	1.00	56/0.23	1.00
Q2	160/0.70	0.99 (0.79–1.24)	109/0.48	1.05 (0.80–1.39)	51/0.22	0.88 (0.60–1.28)
Q3	186/0.89	1.17 (0.95–1.45)	114/0.55	1.12 (0.85–1.47)	72/0.35	1.27 (0.89–1.80)
Q4	150/0.83	1.00 (0.80–1.26)	100/0.55	1.04 (0.78–1.37)	50/0.28	0.94 (0.64–1.38)
Q5	193/1.06	1.15 (0.93–1.43)	120/0.66	1.11 (0.84–1.44)	73/0.40	1.23 (0.86–1.75)
P_trend_^a^		0.22		0.54		0.23
BMI (kg/m^2^)						
Q1	164/0.71	1.00	113/0.49	1.00	51/0.22	1.00
Q2	184/0.82	1.12 (0.90–1.38)	106/0.48	0.94 (0.72–1.22)	78/0.35	1.50 (1.06–2.14)
Q3	158/0.84	1.14 (0.92–1.42)	105/0.56	1.08 (0.83–1.41)	53/0.28	1.27 (0.86–1.87)
Q4	183/0.87	1.10 (0.89–1.37)	111/0.53	0.98 (0.75–1.28)	72/0.34	1.37 (0.95–1.97)
Q5	154/0.82	1.05 (0.84–1.31)	106/0.57	1.04 (0.80–1.36)	48/0.26	1.07 (0.72–1.59)
P_trend_^a^		0.70		0.68		0.94
TFMI (kg/m^2^)						
Q1	159/0.65	1.00	103/0.42	1.00	56/0.23	1.00
Q2	166/0.72	1.00 (0.81–1.25)	109/0.47	1.02 (0.78–1.33)	57/0.25	0.98 (0.68–1.42)
Q3	178/0.86	1.12 (0.90–1.39)	108/0.52	1.04 (0.79–1.37)	70/0.34	1.26 (0.88–1.79)
Q4	164/0.88	1.08 (0.87–1.35)	112/0.60	1.12 (0.86–1.47)	52/0.28	0.99 (0.68–1.46)
Q5	176/1.03	1.13 (0.90–1.41)	109/0.64	1.07 (0.81–1.41)	67/0.39	1.24 (0.86–1.78)
P_trend_^a^		0.21		0.45		0.29
Trunk fat %						
Q1	162/0.67	1.00	106/0.44	1.00	56/0.23	1.00
Q2	164/0.69	0.92 (0.74–1.15)	102/0.43	0.89 (0.68–1.17)	62/0.26	1.06 (0.73–1.52)
Q3	181/0.88	1.12 (0.90–1.38)	120/0.58	1.14 (0.87–1.48)	61/0.30	1.12 (0.78–1.61)
Q4	159/0.85	1.02 (0.82–1.28)	102/0.55	0.99 (0.75–1.30)	57/0.31	1.08 (0.75–1.58)
Q5	177/1.04	1.12 (0.90–1.40)	111/0.65	1.07 (0.81–1.40)	66/0.39	1.25 (0.87–1.80)
P_trend_^a^		0.20		0.45		0.25
Per SD increase						
Trunk fat to trunk fat free mass						
Q1	160/0.67	1.00	104/0.44	1.00	56/0.24	1.00
Q2	159/0.68	0.91 (0.73–1.14)	103/0.44	0.91 (0.69–1.19)	56/0.24	0.95 (0.65–1.37)
Q3	181/0.88	1.10 (0.89–1.37)	118/0.57	1.11 (0.85–1.45)	63/0.30	1.13 (0.79–1.62)
Q4	162/0.86	1.01 (0.81–1.25)	102/0.54	0.98 (0.74–1.29)	60/0.32	1.10 (0.76–1.60)
Q5	181/1.04	1.10 (0.88–1.36)	114/0.65	1.07 (0.82–1.41)	67/0.38	1.22 (0.85–1.76)
P_trend_^a^		0.17		0.48		0.18
Leg fat mass (kg)						
Q1	216/0.99	1.00	117/0.44	1.00	73/0.27	1.00
Q2	163/0.82	1.01 (0.82–1.24)	115/0.51	1.07 (0.83–1.39)	59/0.26	0.90 (0.64–1.27)
Q3	176/0.85	1.05 (0.85–1.29)	108/0.52	1.05 (0.81–1.37)	64/0.31	1.03 (0.74–1.45)
Q4	159/0.77	0.99 (0.79–1.23)	95/0.54	1.04 (0.79–1.37)	48/0.27	0.89 (0.61–1.29)
Q5	129/0.60	1.08 (0.86–1.34)	106/0.63	1.14 (0.86–1.50)	58/0.34	0.98 (0.68–1.41)
P_trend_^a^		0.62		0.47		0.91
Per SD increase						
Ratio of trunk fat mass to leg fat mass						
Q1	157/0.65	1.00	103/0.45	1.00	54/0.24	1.00
Q2	161/0.66	0.97 (0.77–1.20)	105/0.47	0.96 (0.71–1.23)	56/0.25	0.98 (0.67–1.42)
Q3	179/0.89	1.06 (0.85–1.32)	123/0.56	1.09 (0.83–1.42)	60/0.27	0.99 (0.69–1.44)
Q4	159/0.85	1.08 (0.86–1.34)	102/0.50	0.95 (0.73–1.28)	73/0.36	1.31 (0.91–1.88)
Q5	187/1.12	1.25 (0.99–1.58)	108/0.65	1.22 (0.82–1.44)	59/0.35	1.32 (0.89–1.94)
P_trend_^a^		0.04		0.26		0.05
WC (cm)						
Q1	183/0.65	1.00	114/0.41	1.00	69/0.25	1.00
Q2	168/0.71	1.07 (0.86–1.32)	107/0.45	1.06 (0.81–1.38)	61/0.26	1.08 (0.76–1.52)
Q3	165/0.84	1.16 (0.94–1.43)	108/0.55	1.21 (0.93–1.58)	57/0.29	1.07 (0.75–1.52)
Q4	173/1.05	1.34 (1.09–1.66)	108/0.65	1.36 (1.04–1.78)	65/0.39	1.31 (0.92–1.85)
Q5	154/0.94	1.16 (0.93–1.45)	104/0.64	1.23 (0.93–1.62)	50/0.31	1.04 (0.71–1.53)
P_trend_^a^		0.03		0.03		0.45
WHR						
Q1	155/0.63	1.00	95/0.39	1.00	60/0.24	1.00
Q2	148/0.67	0.99 (0.79–1.25)	99/0.45	1.09 (0.82–1.44)	49/0.22	0.85 (0.58–1.24)
Q3	186/0.90	1.24 (1.00–1.54)	115/0.55	1.25 (0.95–1.65)	71/0.34	1.22 (0.87–1.73)
Q4	175/0.93	1.24 (0.99–1.54)	114/0.61	1.30 (0.99–1.71)	61/0.32	1.15 (0.80–1.64)
Q5	179/1.00	1.24 (0.99–1.54)	118/0.66	1.31 (0.99–1.72)	61/0.34	1.12 (0.78–1.61)
P_trend_^a^		0.001		0.02		0.23

All models were adjusted for age at enrollment, education, physical activity, alcohol intake, smoking, history of diabetes, height, red meat intake, processed meat intake, fruits and vegetable intake, folate supplement intake, Vitamin D supplement intake.

Ranges:

Men: FMI: ≤ 3.7, 3.8–4.5, 4.6–5.1, 5.2–5.7, >5.7; body fat %: ≤16.4, 16.5–19.2, 19.3–21.4, 21.5–23.7, >23.7; whole body fat to whole body fat free mass: ≤0.20, 0.21–0.24, 0.25–0.27, 0.28–0.31, >0.31; BMI: ≤ 22.1, 22.2–23.3, 23.4–23.8, 23.9–24.5, >24.5; TFMI: ≤ 2.22; 2.23–2.81, 2.82–3.25, 3.25–3.68, >3.68; trunk fat %: ≤17.2, 17.3–20.9, 21.0–23.7, 23.8–26.6, >26.6; trunk fat mass to trunk fat free mass: ≤0.20, 0.21–0.26, 0.27–0.31, 0.31–0.36, >0.36; leg fat mass: ≤3.3, 3.4–3.8, 3.9–4.2, 4.3–4.6, >4.6; ratio of trunk fat mass to leg fat mass: ≤1.93, 1.94–2.21, 2.22–2.44, 2.45–2.69, >2.69; WC: ≤ 82, 83–85, 86–88, 89–92, >92; WHR: ≤ 0.85, 0.86–0.88, 0.89–0.91, 0.92–0.94, >0.94 for quintiles 1,2 3, 4, and 5, respectively.

Women: FMI: ≤ 6.0, 6.1–6.9, 7.0–7.6, 7.7–8.4, >8.4; body fat %: ≤27.4, 27.5–30.5, 30.6–32.8, 32.9–35.1, >35.1; whole body fat to whole body fat free mass: ≤0.38, 0.39–0.44, 0.45–0.49, 0.50–0.54,> 0.54; BMI: ≤ 21.4, 21.5–22.6, 22.7–23.5, 23.6–24.2, >24.2; TFMI: ≤ 2.86; 2.87–3.44, 3.45–3.90, 3.91–4.39, >4.39; trunk fat %: ≤23.8, 23.9–27.8, 27.9–30.6, 30.7–33.6, >33.6; trunk fat mass to trunk fat free mass: ≤0.31, 0.32–0.38, 0.39–0.44, 0.45–0.50, >0.50; leg fat mass: ≤6.8, 6.9–7.6, 7.7–8.2, 8.3–8.8, >8.8; ratio of trunk fat mass to leg fat mass: ≤1.06, 1.07–1.19, 1.20–1.30, 1.31–1.42, >1.43; WC: ≤ 70, 71–74, 75–77, 78–80.3, >80.3; WHR: ≤ 0.74, 0.75–0.77, 0.78–0.80, 0.81–0.83, >0.83 for quintiles 1,2 3, 4, and 5, respectively.

*PY* person-year, *CI* confidence interval, *HR* hazard ratio, *SD* standard deviation, *FMI* fat mass index, *TFMI* trunk fat mass index.

^a^All tests were two-sided.

Generally, the HRs per SD increase in the anthropometric measures were similar in magnitude to those for the BIA measures (Table 3, Supplementary Tables 3, 4).

The associations between the body measures and risk of the outcomes in models additionally adjusted for BMI are shown in Supplementary Table 2 and Supplementary Tables 5–7. When considering the standard definitions for WC and WHR (women only), adjustment for BMI attenuated the associations, but WC remained associated with risk of postmenopausal breast cancer and postmenopausal endometrial cancer while WHR remained associated with endometrial cancer (overall and among postmenopausal women) (Supplementary Table 2). For our analyses based on quintiles of the BIA-derived exposures, after adjustment for BMI, all of the observed associations with risk of postmenopausal breast cancer and endometrial cancer disappeared (Supplementary Table 5). Among men, adjusting for BMI strengthened the observed associations of the body measures with risk of colorectal cancer (Supplementary Tables 2 and 7).

After exclusion of participants with a follow-up time of 2 years or less (Table 5), the observed associations between the body fat measures and risk of postmenopausal breast cancer were attenuated, but all associations (except that for WHR) were statistically significant. The observed associations between the body fat measures and risk of endometrial cancer and male colorectal cancer disappeared in analyses among participants who were followed up for more than two years (Table 5 and Supplementary Table 8). There were no associations between the body fat measures and risk of ovarian cancer and colorectal cancer (men and women combined or women only) after exclusion of participants with follow-up time of 2 years or less (Tables 5 and 6 and Supplementary Table 8).

**Table 5:** Hazard ratios and 95% CI for the association of baseline BIA-derived measures of body fat with risk of incident, invasive female-specific cancers among women in the UK Biobank (excluding participants within two years of recruitment).

	Breast (postmenopausal)^a^	Endometrium	Ovary
	Multivariable-adjusted HR (95% CI)		
FMI (kg/m^2^)			
Q1	1.00	1.00	1.00
Q2	1.11 (0.87–1.42)	1.38 (0.77–2.49)	1.17 (0.69–1.97)
Q3	1.21 (0.95–1.54)	1.03 (0.54–1.97)	0.65 (0.35–1.21)
Q4	1.19 (0.93–1.51)	1.52 (0.84–2.75)	1.28 (0.76–2.15)
Q5	1.46 (1.16–1.84)	1.35 (0.72–2.56)	0.71 (0.38–1.33)
P_trend_^b^	0.001	0.32	0.49
Per SD increase	1.15 (1.06–1.23)	1.14 (0.96–1.34)	0.98 (0.83–1.15)
Body fat %			
Q1	1.00	1.00	1.00
Q2	1.05 (0.82–1.35)	1.30 (0.72–2.34)	0.78 (0.46–1.34)
Q3	1.23 (0.97–1.57)	1.08 (0.57–2.04)	0.61 (0.34–1.11)
Q4	1.23 (0.97–1.57)	1.31 (0.70–2.45)	1.19 (0.72–1.99)
Q5	1.33 (1.05–1.69)	1.43 (0.76–2.69)	0.61 (0.33–1.12)
P_trend_^b^	0.007	0.33	0.50
Per SD increase	1.13 (1.05–1.23)	1.13 (0.95–1.34)	0.98 (0.83–1.16)
Whole body fat mass to whole body fat free mass			
Q1	1.00	1.00	1.00
Q2	1.09 (0.85–1.40)	1.29 (0.71–2.32)	0.80 (0.47–1.38)
Q3	1.23 (0.97–1.57)	0.96 (0.50–1.85)	0.63 (0.35–1.14)
Q4	1.27 (0.99–1.62)	1.44 (0.78–2.66)	1.28 (0.77–2.13)
Q5	1.35 (1.06–1.71)	1.37 (0.73–2.57)	0.60 (0.32–1.11)
P_trend_^b^	0.006	0.31	0.56
Per SD increase	1.13 (1.04–1.22)	1.12 (0.95–1.32)	0.98 (0.83–1.15)
BMI (kg/m^2^)			
Q1	1.00	1.00	1.00
Q2	1.27 (1.01–1.61)	0.86 (0.46–1.59)	0.66 (0.38–1.16)
Q3	1.23 (0.97–1.56)	1.18 (0.67–2.11)	0.87 (0.52–1.46)
Q4	1.29 (1.02–1.64)	0.94 (0.50–1.75)	0.75 (0.43–1.30)
Q5	1.46 (1.16–1.84)	1.28 (0.72–2.28)	0.75 (0.44–1.30)
P_trend_^b^	0.003	0.39	0.43
Per SD increase	1.12 (1.04–1.20)	1.13 (0.96–1.33)	0.97 (0.83–1.14)
TFMI (kg/m^2^)			
Q1	1.00	1.00	1.00
Q2	0.98 (0.77–1.25)	1.33 (0.72–2.43)	0.69 (0.40–1.20)
Q3	1.16 (0.92–1.48)	1.17 (0.62–2.24)	0.67 (0.38–1.19)
Q4	1.07 (0.84–1.37)	1.25 (0.65–2.39)	1.06 (0.63–1.77)
Q5	1.47 (1.16–1.85)	1.87 (0.99–3.50)	0.67 (0.36–1.22)
P_trend_^b^	0.001	0.10	0.60
Per SD increase	1.12 (0.99–1.25)	1.16 (0.98–1.37)	0.97 (0.82–1.14)
Trunk fat %			
Q1	1.00	1.00	1.00
Q2	1.05 (0.82–1.34)	0.90 (0.49–1.65)	0.56 (0.31–1.01)
Q3	1.16 (0.90–1.48)	1.01 (0.54–1.88)	1.02 (0.60–1.73)
Q4	1.17 (0.92–1.50)	1.16 (0.62–2.16)	1.18 (0.70–1.99)
Q5	1.48 (1.16–1.88)	1.59 (0.86–2.94)	0.59 (0.31–1.13)
P_trend_^b^	0.001	0.095	0.83
Per SD increase	1.14 (1.05–1.23)	1.11 (0.90–1.39)	0.97 (0.82–1.15)
Trunk fat mass to trunk fat free mass			
Q1	1.00	1.00	1.00
Q2	1.00 (0.78–1.28)	0.87 (0.47–1.61)	0.55 (0.31–0.99)
Q3	1.16 (0.91–1.48)	0.92 (0.49–1.74)	0.94 (0.55–1.60)
Q4	1.15 (0.90–1.48)	1.14 (0.61–2.14)	1.16 (0.69–1.96)
Q5	1.43 (1.12–1.82)	1.58 (0.86–2.91)	0.60 (0.31–1.13)
P_trend_^b^	0.001	0.090	0.81
Per SD increase	1.13 (1.05–1.21)	1.14 (0.97–1.35)	0.97 (0.82–1.14)
Leg fat mass (kg)			
Q1	1.00	1.00	1.00
Q2	1.15 (0.91–1.45)	0.78 (0.42–1.42)	0.73 (0.43–1.23)
Q3	1.13 (0.89–1.42)	1.13 (0.65–1.96)	0.76 (0.45–1.28)
Q4	1.21 (0.96–1.53)	1.03 (0.57–1.87)	0.53 (0.29–0.99)
Q5	1.33 (1.04–1.70)	1.09 (0.58–2.07)	1.06 (0.60–1.85)
P_trend_^b^	0.023	0.57	0.60
Per SD increase	1.13 (1.04–1.22)	1.07 (0.87–1.31)	0.94 (0.78–1.14)
Ratio of trunk fat mass to leg fat mass			
Q1	1.00	1.00	1.00
Q2	1.12 (0.88–1.42)	1.27 (0.69–2.33)	0.46 (0.25–0.85)
Q3	1.10 (0.86–1.40)	0.93 (0.47–1.84)	0.86 (0.51–1.47)
Q4	0.99 (0.76–1.29)	1.27 (0.65–2.49)	0.96 (0.56–1.65)
Q5	1.50 (1.14–1.96)	1.83 (0.90–3.72)	0.72 (0.40–1.30)
P_trend_^b^	0.025	0.15	0.99
Per SD increase	1.12 (1.02–1.22)	1.13 (1.03–1.24)	0.96 (0.77–1.20)
WC (cm)			
Q1	1.00	1.00	1.00
Q2	0.97 (0.78–1.21)	1.68 (0.97–2.90)	0.58 (0.34–0.99)
Q3	1.08 (0.86–1.36)	1.15 (0.61–2.20)	1.13 (0.70–1.84)
Q4	1.22 (0.96–1.55)	1.28 (0.65–2.55)	0.81 (0.45–1.46)
Q5	1.27 (1.02–1.59)	1.53 (0.82–2.86)	0.62 (0.86–1.12)
P_trend_^b^	0.006	0.455	0.36
Per SD increase	1.13 (1.05–1.22)	1.17 (0.99–1.38)	0.97 (0.82–1.14)
WHR			
Q1	1.00	1.00	1.00
Q2	1.10 (0.87–1.38)	0.94 (0.52–1.70)	1.21 (0.71–2.04)
Q3	1.08 (0.86–1.37)	1.43 (0.83–2.48)	0.62 (0.33–1.17)
Q4	1.09 (0.86–1.38)	0.78 (0.41–1.50)	1.38 (0.82–2.30)
Q5	1.24 (0.99–1.56)	1.14 (0.62–2.08)	0.63 (0.33–1.18)
P_trend_^b^	0.096	0.860	0.37
Per SD increase	1.07 (0.99–1.15)	1.14 (0.98–1.34)	0.93 (0.79–1.10)

All models were adjusted for age at enrollment, education, physical activity, alcohol intake, smoking, height. age at menarche, age at first full-term birth and parity combined, HRT status, age at menopause.

Ranges: FMI: ≤ 6.0, 6.1–6.9, 7.0–7.6, 7.7–8.4, >8.4; body fat %: ≤27.4, 27.5–30.5, 30.6–32.8, 32.9–35.1, >35.1; whole body fat to whole body fat free mass: ≤0.38, 0.39–0.44, 0.45–0.49, 0.50–0.54,> 0.54; BMI: ≤ 21.4, 21.5–22.6, 22.7–23.5, 23.6–24.2, >24.2; TFMI: ≤ 2.86; 2.87–3.44, 3.45–3.90, 3.91–4.39, >4.39; trunk fat %: ≤23.8, 23.9–27.8, 27.9–30.6, 30.7–33.6, >33.6; trunk fat mass to trunk fat free mass: ≤0.31, 0.32–0.38, 0.39–0.44, 0.45–0.50, >0.50; leg fat mass: ≤6.8, 6.9–7.6, 7.7–8.2, 8.3–8.8, >8.8; ratio of trunk fat mass to leg fat mass: ≤1.06, 1.07–1.19, 1.20–1.30, 1.31–1.42, >1.43; WC: ≤ 70, 71–74, 75–77, 78–80.3, >80.3; WHR: ≤ 0.74, 0.75–0.77, 0.78–0.80, 0.81–0.83, >0.83 for quintiles 1,2 3, 4 and 5, respectively.

*HR* hazard ratio, *CI* confidence interval, *BMI* body mass index, *WC* waist circumference, *WHR* waist to hip ratio, *FMI* fat mass index, *TFMI* trunk fat mass index.

^a^Also adjusted for family history of breast cancer and mammogram ever.

^b^All tests were two-sided.

**Table 6:** Hazard ratios and 95% CI for the association of baseline BIA-derived measures of body fat with risk of incident, invasive colorectal cancer among participants in the UK Biobank (excluding participants within two years of recruitment).

	Colorectal	Colon	Rectal
FMI (kg/m^2^)			
Q1	1.00	1.00	1.00
Q2	1.03 (0.80–1.32)	1.06 (0.78–1.45)	0.96 (0.62–1.47)
Q3	1.04 (0.81–1.33)	1.09 (0.80–1.48)	0.94 (0.61–1.45)
Q4	1.07 (0.83–1.38)	0.99 (0.73–1.37)	1.21 (0.80–1.83)
Q5	1.15 (0.90–1.48)	1.08 (0.79–1.48)	1.30 (0.86–1.96)
P_trend_^a^	0.24	0.78	0.11
Body fat %			
Q1	1.00	1.00	1.00
Q2	0.96 (0.74–1.23)	1.06 (0.78–1.44)	0.78 (0.50–1.21)
Q3	1.09 (0.85–1.39)	1.07 (0.78–1.46)	1.12 (0.74–1.69)
Q4	0.99 (0.76–1.28)	1.01 (0.73–1.40)	0.94 (0.61–1.46)
Q5	1.16 (0.90–1.48)	1.08 (0.79–1.48)	1.30 (0.86–1.95)
P_trend_^a^	0.25	0.77	0.13
Whole body fat mass to whole body fat free mass			
Q1	1.00	1.00	1.00
Q2	0.94 (0.73–1.21)	1.04 (0.77–1.42)	0.76 (0.49–1.19)
Q3	1.06 (0.82–1.36)	1.03 (0.75–1.41)	1.10 (0.73–1.66)
Q4	0.95 (0.73–1.24)	0.99 (0.71–1.37)	0.89 (0.57–1.39)
Q5	1.14 (0.89–1.46)	1.07 (0.78–1.46)	1.26 (0.84–1.89)
P_trend_^a^	0.32	0.82	0.17
BMI (kg/m^2^)			
Q1	1.00	1.00	1.00
Q2	1.09 (0.86–1.39)	0.89 (0.66–1.21)	1.56 (1.03–2.36)
Q3	1.09 (0.84–1.40)	1.09 (0.81–1.48)	1.05 (0.65–1.68)
Q4	1.09 (0.85–1.39)	0.91 (0.67–1.23)	1.50 (0.99–2.27)
Q5	1.02 (0.79–1.31)	0.96 (0.70–1.30)	1.16 (0.73–1.82)
P_trend_^a^	0.90	0.84	0.64
TFMI (kg/m^2^)			
Q1	1.00	1.00	1.00
Q2	0.99 (0.77–1.27)	1.06 (0.79–1.44)	0.85 (0.55–1.31)
Q3	0.99 (0.77–1.28)	0.92 (0.67–1.27)	1.12 (0.74–1.69)
Q4	0.98 (0.75–1.26)	1.05 (0.77–1.44)	0.83 (0.53–1.31)
Q5	1.17 (0.91–1.50)	1.07 (0.78–1.46)	1.36 (0.91–2.04)
P_trend_^a^	0.28	0.76	0.17
Trunk fat %			
Q1	1.00	1.00	1.00
Q2	0.91 (0.71–1.17)	0.91 (0.67–1.24)	0.91 (0.59–1.39)
Q3	1.02 (0.80–1.31)	1.03 (0.76–1.40)	1.00 (0.66–1.53)
Q4	0.99 (0.77–1.28)	1.01 (0.74–1.38)	0.95 (0.61–1.47)
Q5	1.12 (0.87–1.44)	1.01 (0.73–1.39)	1.35 (0.90–2.02)
P_trend_^a^	0.26	0.73	0.16
Trunk fat mass to trunk fat free mass			
Q1	1.00	1.00	1.00
Q2	0.88 (0.68–1.13)	0.91 (0.67–1.24)	0.82 (0.53–1.26)
Q3	0.99 (0.78–1.28)	0.99 (0.73–1.35)	1.01 (0.66–1.53)
Q4	0.98 (0.76–1.26)	0.98 (0.72–1.35)	0.97 (0.63–1.49)
Q5	1.13 (0.88–1.45)	1.03 (0.75–1.41)	1.32 (0.88–1.98)
P_trend_^a^	0.21	0.71	0.11
Leg fat mass (kg)			
Q1	1.00	1.00	1.00
Q2	0.90 (0.71–1.15)	1.04 (0.77–1.39)	0.69 (0.45–1.05)
Q3	1.07 (0.84–1.35)	1.06 (0.78–1.43)	1.09 (0.74–1.59)
Q4	0.88 (0.68–1.14)	0.93 (0.67–1.28)	0.79 (0.51–1.22)
Q5	1.09 (0.85–1.40)	1.12 (0.82–1.54)	1.04 (0.69–1.56)
P_trend_^a^	0.63	0.74	0.72
Ratio of trunk fat mass to leg fat mass			
Q1	1.00	1.00	1.00
Q2	0.99 (0.77–1.28)	0.99 (0.72–1.35)	1.00 (0.66–1.53)
Q3	0.99 (0.77–1.28)	1.13 (0.83–1.53)	0.76 (0.49–1.20)
Q4	1.09 (0.84–1.41)	1.00 (0.73–1.39)	1.24 (0.82–1.87)
Q5	1.16 (0.88–1.52)	1.07 (0.76–1.51)	1.31 (0.85–2.04)
P_trend_^a^	0.22	0.70	0.12
WC (cm)			
Q1	1.00	1.00	1.00
Q2	1.01 (0.80–1.29)	0.97 (0.72–1.31)	1.11 (0.73–1.67)
Q3	1.17 (0.92–1.49)	1.06 (0.79–1.44)	1.37 (0.92–2.04)
Q4	1.21 (0.94–1.55)	1.17 (0.86–1.60)	1.27 (0.84–1.93)
Q5	1.12 (0.86–1.45)	1.13 (0.83–1.55)	1.08 (0.69–1.69)
P_trend_^a^	0.16	0.23	0.48
WHR			
Q1	1.00	1.00	1.00
Q2	0.94 (0.72–1.22)	1.04 (0.76–1.44)	0.77 (0.49–1.21)
Q3	1.27 (0.99–1.62)	1.23 (0.90–1.68)	1.33 (0.90–1.97)
Q4	1.17 (0.91–1.51)	1.26 (0.92–1.72)	1.02 (0.66–1.57)
Q5	1.21 (0.94–1.56)	1.21 (0.88–1.66)	1.21 (0.80–1.84)
P_trend_^a^	0.04	0.12	0.19

All models were adjusted for age at enrollment, education, physical activity, alcohol intake, smoking, height, history of diabetes, red meat intake, processed meat intake, fruits and vegetable intake, folate supplement intake, Vitamin D supplement intake.

^a^All tests were two-sided.

Ranges:

Men: FMI: ≤ 3.7, 3.8–4.5, 4.6–5.1, 5.2–5.7, >5.7; body fat %: ≤16.4, 16.5–19.2, 19.3–21.4, 21.5–23.7, >23.7; whole body fat to whole body fat free mass: ≤0.20, 0.21–0.24, 0.25–0.27, 0.28–0.31, >0.31; BMI: ≤ 22.1, 22.2–23.3, 23.4–23.8, 23.9–24.5, >24.5; TFMI: ≤ 2.22; 2.23–2.81, 2.82–3.25, 3.25–3.68, >3.68; trunk fat %: ≤17.2, 17.3–20.9, 21.0–23.7, 23.8–26.6, >26.6; trunk fat mass to trunk fat free mass: ≤0.20, 0.21–0.26, 0.27–0.31, 0.31–0.36, >0.36; leg fat mass: ≤3.3, 3.4–3.8, 3.9–4.2, 4.3–4.6, >4.6; ratio of trunk fat mass to leg fat mass: ≤1.93, 1.94–2.21, 2.22–2.44, 2.45–2.69, >2.69; WC: ≤ 82, 83–85, 86–88, 89–92, >92; WHR: ≤ 0.85, 0.86–0.88, 0.89–0.91, 0.92–0.94, >0.94 for quintiles 1,2 3, 4, and 5, respectively.

Women: FMI: ≤ 6.0, 6.1–6.9, 7.0–7.6, 7.7–8.4, >8.4; body fat %: ≤27.4, 27.5–30.5, 30.6–32.8, 32.9–35.1, >35.1; whole body fat to whole body fat free mass: ≤0.38, 0.39–0.44, 0.45–0.49, 0.50–0.54,> 0.54; BMI: ≤ 21.4, 21.5–22.6, 22.7–23.5, 23.6–24.2, >24.2; TFMI: ≤ 2.86; 2.87–3.44, 3.45–3.90, 3.91–4.39, >4.39; trunk fat %: ≤23.8, 23.9–27.8, 27.9–30.6, 30.7–33.6, >33.6; trunk fat mass to trunk fat free mass: ≤0.31, 0.32–0.38, 0.39–0.44, 0.45–0.50, >0.50; leg fat mass: ≤6.8, 6.9–7.6, 7.7–8.2, 8.3–8.8, >8.8; ratio of trunk fat mass to leg fat mass: ≤1.06, 1.07–1.19, 1.20–1.30, 1.31–1.42, >1.43; WC: ≤ 70, 71–74, 75–77, 78–80.3, >80.3; WHR: ≤ 0.74, 0.75–0.77, 0.78–0.80, 0.81–0.83, >0.83 for quintiles 1,2 3, 4, and 5, respectively.

*HR* hazard ratio, *CI* confidence interval, *BMI* body mass index, *WC* waist circumference, *WHR* waist to hip ratio, *FMI* fat mass index, *TFMI* trunk fat mass index.

^a^All tests were two-sided.

## Discussion

Using BIA-derived and anthropometric measures of adiposity, we showed that general and central/peripheral adiposity were positively associated with risk of invasive breast cancer among normal weight postmenopausal women. These findings are in accord with those of a recent prospective study in which an approximate doubling in the risk of developing invasive breast cancer was observed among normal weight postmenopausal women with relatively high DXA-derived measures of whole body fat mass and trunk fat mass.^5^ Furthermore, in a case-control study, which focused on risk of breast cancer among normal weight women of African descent, those with a relatively high WC (i.e. above the median value of 81 cm), also had increased risk of breast cancer.^25^ In the present study, we also showed that even among normal weight women, central adiposity (i.e. being in the highest quintile for WC > 80.3 cm or WHR > 0.83), but not general adiposity, was positively associated with risk of endometrial cancer, particularly among postmenopausal women. This result is consistent with the findings of a previous study in which central adiposity (WC > 88 cm) was associated with a significant increase in the risk of endometrial cancer among normal weight women.^6^ A recent study conducted in the UK Biobank cohort, which included the entire BMI range, also observed positive associations between WC and WHR with risk of endometrial cancer.^20^ In contrast to the findings for postmenopausal breast cancer and endometrial cancer, we did not observe any association between the body fat measures and risk of ovarian cancer. As suggested by previous studies, it is probable that excess adiposity influences specific histological subtypes of ovarian cancer, namely borderline serous, invasive endometrioid and invasive mucinous tumours.^26^ However, we could not evaluate the associations between the body fat measures and risk of the aforementioned histological subtypes of ovarian tumour due to the relatively small number of cases with each histological subtype.

Excess body fat is considered to be a risk factor for colorectal cancer.^1^ However, in the present study, there was no association between the body fat measures and risk of colorectal cancer among normal weight men and women combined. Existing evidence suggests that the association between body fat and risk of colorectal cancer is stronger among men than women.^27^ In line with this, our study showed relatively weak, but positive associations between some measures of central adiposity and risk of colorectal and colon cancer among men. However, none of the measures was associated with risk of colorectal cancer among women. Based on our review of the literature, we are not aware of any previous studies of the associations of body fat measures with risk of colorectal cancer in normal weight individuals. Therefore, further studies are warranted to confirm our results.

In analyses in which we estimated the HRs per SD in order to make comparisons between the associations for the BIA-derived measures and the anthropometry measures, the associations per SD increase in the BIA-derived measures were comparable in magnitude to those for BMI, suggesting that the BIA-derived measures were not better indicators of risk of the cancers of interest than the anthropometry measures. Further, in models additionally adjusted for BMI, the observed associations of quintiles of BIA-derived measures of general and/or central/peripheral adiposity with risk of breast and endometrial cancers were substantially attenuated. Additionally, in analyses of WC and WHR after categorisation using standard cut points, their positive associations with risk of postmenopausal breast cancer and endometrial cancer (particularly postmenopausal endometrial cancer) were attenuated but remained significant after adjustment for BMI. Among men, the positive association of WC (based on standard cut points) with risk of colorectal cancer was strengthened after adjustment for BMI. These findings imply that BMI only partly explains the association between central adiposity and risk of postmenopausal breast cancer, endometrial cancer and colorectal cancer (males) among normal weight individuals. Our findings, however, should be interpreted with caution, as the proportion of cases with central adiposity based on standard definitions for WC and WHR (i.e. WC ≥ 88 cm or WHR > 0.85 for women and WC ≥ 90 cm or WHR > 0.90 for men) was relatively small. Hence, confirmatory studies with a larger number of events are needed as our findings may be due to chance.

As men and women age, certain physiological changes such as increased adiposity and redistribution of body fat, particularly in the central region, may occur.^28^ This increase in adiposity may increase susceptibility to developing cancer. However, the mechanisms underlying the associations of the body fat measures with risk of postmenopausal breast cancer, endometrial cancer and colorectal (males) among normal weight individuals are incompletely understood. Nevertheless, excess adiposity, particularly central adiposity, is associated with various metabolic perturbations which can promote cancer development. For example, excess adiposity leads to hyperinsulinemia which acts, in turn, to suppress circulating SHBG levels. Reduction of SHBG has been observed in normal BMI postmenopausal women with excess body fat,^5^ and leads to elevated levels of free circulating hormones, including oestrogens and testosterone, which have been linked to tumour cell proliferation and other carcinogenic processes.^4^ Further, insulin can activate the Ras/Raf/MAPK and PI3K/Akt/mTOR pathways that may increase cell proliferation and increase the risk of tumour formation.^4^ Relatively high levels of body fat, particularly in the central region, can also induce inflammatory responses, which can promote cancer development.^29^

Peripheral adiposity is associated with more favourable metabolic health than abdominal adiposity, with greater insulin sensitivity and a more favourable inflammatory profile, and may therefore be associated with lower cancer risk than central adiposity.^30^ However, in the current study and in a previous study among normal weight women from the Women’s Health Initiative cohort,^5^ peripheral adiposity was shown to be associated with increased risk of postmenopausal breast cancer. Of note, fat in this region has been positively correlated with levels of estrone^31^ and leptin,^32,33^ both of which are believed to induce carcinogenesis.^34^ However, further studies are needed to determine whether peripheral body fat can influence breast cancer risk among normal weight individuals.

This is the largest known prospective study to date that has assessed the associations between various body fat measures and risk of obesity-related cancers among normal weight individuals. Further, we were able to assess these associations using direct measurements of body fat composition (i.e. BIA-derived measures) which were made by trained staff using standardised procedures. Although BIA can more reliably assess body composition than anthropometric measures such as BMI, WC and WHR, its accuracy is affected by factors such as the participants’ hydration status, nutritional status, physical activity level and body temperature.^2^ Further, the predictive equations used in BIA assessments are population-specific and device specific, and therefore, BIA is most appropriate in individuals with the same characteristics as those of the reference population.^10^ BIA is also a less accurate method than DXA for determining % body fat, whole body fat mass, and lean body mass among lean individuals.^35,36^ However, in a recent study within the UK Biobank, BIA-derived fat mass was shown to have a strong correlation with DXA-derived fat mass.^37^ Our study has several other limitations. WC and BIA-derived trunk fat do not discriminate between visceral and subcutaneous fat, and therefore, are not ideal markers of visceral fat. For endometrial and ovarian cancer, the number of cases within some strata were relatively small, and, hence, findings for these cancer sites may be due to chance. Given that UK Biobank participants are healthier than the general population,^14^ our findings may not be representative of the general population. Further, for endometrial cancer, colorectal cancer among men, and to a lesser extent, for postmenopausal breast cancer in women, it is possible that the observed associations were partly due to reverse causality as they were attenuated after exclusion of participants with two years or less of follow-up; however, attenuation of the associations may also have been due to loss of power resulting from a reduced sample size. Finally, we were unable to examine the associations between the body fat measures and risk of the cancers of interest by tumour stage and by hormone receptor subtype (for breast cancer) because information on these characteristics is not available in the UK Biobank.

In conclusion, the findings of this study suggest that the current normal weight category based on BMI includes individuals who are at increased risk of some obesity-related cancers. Hence, the current categorisation of individuals as normal weight according to their BMI may need to be re-evaluated in order to better characterise their risk of obesity-related cancers.

## Supplementary information

Supplementary Tables

## Data Availability

This study was conducted using data from the UK Biobank study. Information on data availability can be obtained via the UK Biobank website (http://www.ukbiobank.ac.uk).
