# Supplementary material for: The association of body fat composition with risk of breast, endometrial, ovarian and colorectal cancers among normal weight participants in the UK Biobank
Source: Br J Cancer. 2021 Mar 15;124(9):1592–605. doi: 10.1038/s41416-020-01210-y (PMC8076175; doi:10.1038/s41416-020-01210-y)
Supplement: Supplementary file 1 — Supplementary Tables [file 41416_2020_1210_MOESM1_ESM.docx]

**SUPPLEMENTARY MATERIAL**

**eMethods**

The Townsend deprivation index, which is used as a proxy for socioeconomic status, is a score which corresponds to the output area in which a participant’s postcode is located. To assess the participants’ physical activity level, information on types and frequency of physical activity was collected. Metabolic equivalent (MET)-hours/week were then computed by multiplying the number of hours per week of each leisure-time physical activity by the MET value of the activity and summing over all types of activities. To assess intake of alcohol and the diet-related factors, participants were asked to report their frequency of intake of alcohol, fruits and vegetables, red meat and processed meat intake over the last twelve months. To determine whether participants had a family history of breast or colorectal cancer, participants were asked if any of their biological siblings or biological parents had ever suffered from breast cancer or bowel cancer; To obtain information on history of diabetes, participants were asked, “Has a doctor ever told you that you have diabetes?” With respect to supplement use, participants were asked, "Do you regularly take any of the supplements (including Vitamin D and folate)?”, while for ibuprofen and aspirin use, participants were asked during a nurse-led interview to report the prescription medications which they took regularly. To ascertain age at menarche, women were asked "How old were you when your periods started?", for parity, they were asked "How many children have you given birth to (live children only)?, for age at first live birth, they were asked, "How old were you when you had your first child?", for HRT status (never/former), participants were asked if they had ever used hormone therapy and the age that they last used hormone therapy (current users indicated that they were still taking hormone therapy; former users were those who reported ever using hormone therapy and provided age stopped, never users were those who reported never having used hormone therapy, unknown/missing were those who did not respond to having used hormone therapy and those who indicated that they had used hormone therapy but did not report an age at stopping use). For age at menopause, participants were asked" How old were you when your periods stopped?" and for mammogram screening, participants were asked "Have you ever been for breast cancer screening (a mammogram)?"

**Supplementary Table 1: Hazard ratios and 95% CI for the association of BIA-derived baseline body fat measures with risk of incident, invasive endometrial and ovarian cancers among postmenopausal women in the UK Biobank**

|  | Endometrium  N= 110 | Ovary  N=129 |
| --- | --- | --- |
|  | Multivariable-adjusted HR (95% CI) | Multivariable-adjusted HR (95% CI) |
| **FMI (kg/m^2^)** |  |  |
| Q1 | 1.00 | 1.00 |
| Q2 | 1.04 (0.55-1.94) | 1.36 (0.79-2.36) |
| Q3 | 0.68 (0.34-1.38) | 0.90 (0.49-1.65) |
| Q4 | 1.39 (0.78-2.51) | 1.33 (0.76-2.30) |
| Q5 | 1.44 (0.80-2.57) | 1.08 (0.61-1.93) |
| P_trend_^†^ | 0.10 | 0.85 |
| Per SD increase | 1.20 (0.98-1.47) | 1.01 (0.85-1.21) |
| **Body fat %** |  |  |
| Q1 | 1.00 | 1.00 |
| Q2 | 1.18 (0.62-2.27) | 0.80 (0.45-1.41) |
| Q3 | 0.83 (0.41-1.68) | 0.81 (0.46-1.44) |
| Q4 | 1.36 (0.72-2.58) | 1.17 (0.69-1.99) |
| Q5 | 1.75 (0.95-3.21) | 0.85 (0.48-1.48) |
| P_trend_^†^ | 0.04 | 0.90 |
| Per SD increase | 1.21 (0.98-1.48) | 1.01 (0.84-1.22) |
| **Ratio of whole body fat mass to whole body fat free mass** |  |  |
| Q1 | 1.00 | 1.00 |
| Q2 | 1.17 (0.61-2.25) | 0.82 (0.46-1.47) |
| Q3 | 0.83 (0.41-1.68) | 0.81 (0.45-1.45) |
| Q4 | 1.37 (0.72-2.60) | 1.31 (0.77-2.21) |
| Q5 | 1.67 (0.91-3.07) | 0.84 (0.48-1.49) |
| P_trend_^†^ | 0.74 | 0.80 |
| Per SD increase | 1.05 (0.85-1.31) | 1.01 (0.84-1.21) |
| **BMI (kg/m^2^)** |  |  |
| Q1 | 1.00 | 1.00 |
| Q2 | 0.79 (0.41-1.51) | 0.79 (0.45-1.38) |
| Q3 | 1.31 (0.73-2.32) | 1.09 (0.66-1.83) |
| Q4 | 1.01 (0.54-1.86) | 0.92 (0.54-1.57) |
| Q5 | 1.35 (0.76-2.40) | 0.85 (0.49-1.47) |
| P_trend_^†^ | 0.22 | 0.75 |
| Per SD increase | 1.14 (0.93-1.39) | 1.01 (0.84-1.20) |
| **TFMI (kg/m^2^)** |  |  |
| Q1 | 1.00 | 1.00 |
| Q2 | 1.42 (0.75-2.68) | 0.82 (0.47-1.44) |
| Q3 | 0.74 (0.35-1.56) | 0.80 (0.45-1.41) |
| Q4 | 1.31 (0.68-2.51) | 1.15 (0.68-1.95) |
| Q5 | 2.30 (1.27-4.15) | 0.91 (0.52-1.60) |
| P_trend_^†^ | 0.01 | 0.80 |
| Per SD increase | 1.28 (1.06-1.56) | 1.01 (0.84-1.21) |
| **Trunk fat %** |  |  |
| Q1 | 1.00 | 1.00 |
| Q2 | 0.89 (0.47-1.71) | 0.55 (0.30-1.02) |
| Q3 | 0.71 (0.35-1.44) | 1.00 (0.58-1.72) |
| Q4 | 1.17 (0.62-2.21) | 1.23 (0.73-2.08) |
| Q5 | 1.73 (0.95-3.16) | 0.77 (0.43-1.39) |
| P_trend_^†^ | 0.02 | 0.65 |
| Per SD increase | 1.23 (0.99-1.53) | 1.00 (0.83-1.21) |
| **Ratio of trunk fat mass to trunk fat free mass** |  |  |
| Q1 | 1.00 | 1.00 |
| Q2 | 0.87 (0.46-1.67) | 0.51 (0.27-0.95) |
| Q3 | 0.63 (0.31-1.30) | 0.95 (0.56-1.64) |
| Q4 | 1.21 (0.65-2.26) | 1.21 (0.72-2.05) |
| Q5 | 1.63 (0.90-2.98) | 0.73 (0.42-1.36) |
| P_trend_^†^ | 0.03 | 0.63 |
| Per SD increase | 1.20 (1.01-1.44) | 1.00 (0.83-1.20) |
| **Leg fat mass (kg)** |  |  |
| Q1 | 1.00 | 1.00 |
| Q2 | 0.93 (0.49-1.76) | 1.12 (0.66-1.92) |
| Q3 | 1.19 (0.65-2.18) | 1.28 (0.72-2.27) |
| Q4 | 1.32 (0.72-2.40) | 1.39 (0.74-2.62) |
| Q5 | 1.42 (0.77-2.65) | 1.27 (0.57-2.80) |
| P_trend_^†^ | 0.15 | 0.38 |
| Per SD increase | 1.16 (0.94-1.42) | 1.02 (0.78-1.34) |
| **Ratio of trunk fat mass to leg fat mass** |  |  |
| Q1 | 1.00 | 1.00 |
| Q2 | 0.91 (0.49-1.71) | 0.55 (0.29-1.06) |
| Q3 | 0.65 (0.32-1.32) | 1.18 (0.68-2.04) |
| Q4 | 1.15 (0.60-2.19) | 1.19 (0.66-2.13) |
| Q5 | 1.53 (0.77-3.02) | 1.21 (0.63-2.31) |
| P_trend_^†^ | 0.14 | 0.15 |
| Per SD increase | 1.23 (1.02-1.47) | 1.01 (0.82-1.25) |
| **WC (cm)** |  |  |
| Q1 | 1.00 | 1.00 |
| Q2 | 1.32 (0.74-2.36) | 0.69 (0.41-1.17) |
| Q3 | 0.74 (0.36-1.52) | 1.10 (0.67-1.82) |
| Q4 | 1.23 (0.63-2.40) | 0.83 (0.46-1.48) |
| Q5 | 1.93 (1.09-3.41) | 0.79 (0.46-1.37) |
| P_trend_^†^ | 0.04 | 0.66 |
| Per SD increase | 1.26 (1.05-1.52) | 0.97 (0.81-1.15) |
| **WHR** |  |  |
| Q1 | 1.00 | 1.00 |
| Q2 | 0.77 (0.40-1.46) | 1.52 (0.90-2.58) |
| Q3 | 1.18 (0.66-2.10) | 0.97 (0.54-1.74) |
| Q4 | 0.85 (0.45-1.59) | 1.30 (0.76-2.24) |
| Q5 | 1.43 (0.82-2.50) | 0.72 (0.38-1.34) |
| P_trend_^†^ | 0.20 | 0.25 |
| Per SD increase | 1.25 (1.05-1.50) | - 1. (0.78-1.11) |

- All models were adjusted for age at enrollment, education, age at menarche, age at first full-term birth and parity combined, HRT status, age at menopause, physical activity, height, alcohol intake, smoking. CI= confidence interval; HR= hazard ratio; SD= standard deviation; WC= waist circumference; WHR= waist to hip ratio
- ^†^All tests were two-sided

Ranges:

- FMI: ≤6.0, 6.1-6.9, 7.0-7.6, 7.7-8.4, >8.4; body fat %: ≤27.4, 27.5-30.5, 30.6-32.8, 32.9-35.1, >35.1; whole body fat to whole body fat free mass: ≤0.38, 0.39-0.44, 0.45-0.49, 0.50-0.54,> 0.54; BMI: ≤21.4, 21.5-22.6, 22.7-23.5, 23.6-24.2, >24.2; TFMI: ≤2.86; 2.87-3.44, 3.45-3.90, 3.91-4.39, >4.39; trunk fat %: ≤23.8, 23.9-27.8, 27.9-30.6, 30.7-33.6, >33.6; trunk fat mass to trunk fat free mass: ≤0.31, 0.32-0.38, 0.39-0.44, 0.45-0.50, >0.50; leg fat mass: ≤6.8, 6.9-7.6, 7.7-8.2, 8.3-8.8, >8.8; ratio of trunk fat mass to leg fat mass: ≤1.06, 1.07-1.19, 1.20-1.30, 1.31-1.42, >1.43; WC: ≤70, 71-74, 75-77, 78-80.3, >80.3; WHR: ≤0.74, 0.75-0.77, 0.78-0.80, 0.81-0.83, >0.83 for quintiles 1,2 3, 4, and 5, respectively

**Supplementary Table 2: Associations of waist circumference and waist to hip ratio (based on established cutpoints) with risk of breast (postmenopausal), endometrial, ovarian and colorectal cancer among participants in the UK Biobank**

|  | **No. of cases** | **HR (95% CI)** | **HR (95% CI) ^‡^** |
| --- | --- | --- | --- |
| **Breast cancer (postmenopausal) *,**^†^ |  |  |  |
| **WC (cm)** |  |  |  |
| <80 | 741 | 1.00 | 1.00 |
| ≥80-<88 | 257 | 1.20 (1.03-1.38) | 1.08 (0.93-1.27) |
| ≥88 | 53 | 1.53 (1.16-2.03) | 1.35 (1.01-1.81) |
| P_trend_ |  | <0.001 | 0.054 |
| **WHR** |  |  |  |
| <0.80 | 624 | 1.00 | 1.00 |
| ≥80-≤0.85 | 262 | 1.04 (0.90-1.21) | 0.99 (0.86-1.15) |
| >0.85 | 165 | 1.14 (0.96-1.36) | 1.06 (0.89-1.26) |
| P_trend_ |  | 0.14 | 0.63 |
|  |  |  |  |
| **Endometrial cancer*** |  |  |  |
| **WC (cm)** |  |  |  |
| **All women** |  |  |  |
| <80 | 115 | 1.00 | 1.00 |
| ≥80-<88 | 32 | 1.15 (0.77-1.72) | 1.06 (0.70-1.63) |
| ≥88 | 8 | 1.93 (0.93-4.00) | 1.75 (0.82-3.70) |
| P_trend_ |  | 0.12 | 0.28 |
| **Postmenopausal women** |  |  |  |
| <80 | 73 | 1.00 | 1.00 |
| ≥80-<88 | 29 | 1.43 (0.92-2.22) | 1.35 (0.85-2.18) |
| ≥88 | 8 | 2.47 (1.17-5.22) | 2.31 (1.06-5.04) |
| P_trend_ |  | 0.01 | 0.03 |
|  |  |  |  |
| **WHR** |  |  |  |
| **All women** |  |  |  |
| <0.80 | 102 | 1.00 | 1.00 |
| ≥80-≤0.85 | 23 | 0.66 (0.42-1.03) | 0.63 (0.40-0.99) |
| >0.85 | 30 | 1.64 (1.08-2.49) | 1.54 (1.01-2.36) |
| P_trend_ |  | 0.19 | 0.31 |
| **Postmenopausal women** |  |  |  |
| <0.80 | 63 | 1.00 | 1.00 |
| ≥80-≤0.85 | 20 | 0.83 (0.50-1.38) | 0.80 (0.48-1.34) |
| >0.85 | 27 | 2.02 (1.28-3.19) | 1.91 (1.19-3.06) |
| P_trend_ |  | 0.02 | 0.04 |
|  |  |  |  |
| **Ovarian cancer*** |  |  |  |
| **WC (cm)** |  |  |  |
| **All women** |  |  |  |
| <80 | 134 | 1.00 | 1.00 |
| ≥80-<88 | 29 | 0.86 (0.57-1.29) | 0.85 (0.55-1.31) |
| ≥88 | 7 | 1.39 (0.64-3.01) | 1.37 (0.62-3.03) |
| P_trend_ |  | 0.99 | 0.98 |
| **Postmenopausal women** |  |  |  |
| <80 | 100 | 1.00 | 1.00 |
| ≥80-<88 | 22 | 0.78 (0.49-1.24) | 0.76 (0.46-1.24) |
| ≥88 | 7 | 1.53 (0.70-3.34) | 1.49 (0.67-3.33) |
| P_trend_ |  | 0.96 | 0.92 |
| **WHR** |  |  |  |
| **All women** |  |  |  |
| <0.80 | 110 | 1.00 | 1.00 |
| ≥80-≤0.85 | 40 | 0.96 (0.67-1.39) | 0.96 (0.66-1.39) |
| >0.85 | 20 | 0.85 (0.53-1.38) | 0.85 (0.52-1.39) |
| P_trend_ |  | 0.53 | 0.52 |
| **Postmenopausal women** |  |  |  |
| <0.80 | 85 | 1.00 | 1.00 |
| ≥80-≤0.85 | 28 | 0.80 (0.52-1.24) | 0.79 (0.51-1.23) |
| >0.85 | 16 | 0.79 (0.46-1.35) | 0.77 (0.45-1.34) |
| P_trend_ |  | 0.26 | 0.24 |
|  |  |  |  |
| **WC (cm; Men only)** |  |  |  |
| **Colorectal cancer**^ǁ^ |  |  |  |
| <90 | 227 | 1.00 | 1.00 |
| ≥90 | 130 | 1.30 (1.04-1.63) | 1.39 (1.08-1.79) |
| **Colon**^ǁ^ |  |  |  |
| <90 | 127 | 1.00 | 1.00 |
| ≥90 | 72 | 1.26 (0.93-1.71) | 1.25 (0.90-1.75) |
| **Rectal**^ǁ^ |  |  |  |
| <90 | 100 | 1.00 | 1.00 |
| ≥90 | 58 | 1.34 (0.95-1.88) | 1.58 (1.09-2.31) |
|  |  |  |  |
| **WHR (Men only)** |  |  |  |
| **Colorectal**^ǁ^ |  |  |  |
| <0.90 | 192 | 1.00 | 1.00 |
| ≥0.90 | 165 | 1.23 (0.99-1.52) | 1.26 (1.01-1.57) |
| **Colon**^ǁ^ |  |  |  |
| <0.90 | 107 | 1.00 | 1.00 |
| ≥0.90 | 92 | 1.25 (0.94-1.66) | 1.24 (0.92-1.66) |
| **Rectal**^ǁ^ |  |  |  |
| <0.90 | 85 | 1.00 | 1.00 |
| ≥0.90 | 73 | 1.20 (0.87-1.65) | 1.28 (0.92-1.79) |
|  |  |  |  |
| **WC (cm; women only)** |  |  |  |
| **Colorectal cancer**^ǁ^ |  |  |  |
| <80 | 377 | 1.00 | 1.00 |
| ≥80-<88 | 97 | 0.96 (0.76-1.20) | 0.92 (0.72-1.17) |
| ≥88 | 12 | 0.77 (0.44-1.39) | 0.77 (0.41-1.33) |
| P_trend_ |  | 0.43 | 0.27 |
| **Colon**^ǁ^ |  |  |  |
| <80 | 261 | 1.00 | 1.00 |
| ≥80-<88 | 73 | 1.03 (0.79-1.35) | 1.14 (0.95-1.38) |
| ≥88 | 8 | 0.75 (0.37-1.52) | 1.07 (0.80-1.45) |
| P_trend_ |  | 0.76 | 0.29 |
| **Rectal**^ǁ^ |  |  |  |
| <80 | 116 | 1.00 | 1.00 |
| ≥80-<88 | 24 | 0.79 (0.50-1.23) | 0.70 (0.44-1.11) |
| ≥88 | 4 | 0.86 (0.31-2.36) | 0.74 (0.27-2.05) |
| P_trend_ |  | 0.35 | 0.15 |
|  |  |  |  |
| **WHR (women only)** |  |  |  |
| **Colorectal**^ǁ^ |  |  |  |
| <0.80 | 292 | 1.00 | 1.00 |
| ≥0.80-≤0.85 | 129 | 1.13 (0.92-1.39) | 1.12 (0.90-1.38) |
| >0.85 | 65 | 0.99 (0.76-1.31) | 0.98 (0.74-1.29) |
| P_trend_ |  | 0.68 | 0.79 |
| **Colon**^ǁ^ |  |  |  |
| <0.80 | 205 | 1.00 | 1.00 |
| ≥0.80-≤0.85 | 86 | 1.06 (0.82-1.37) | 1.06 (0.82-1.37) |
| >0.85 | 51 | 1.10 (0.80-1.50) | 1.10 (0.80-1.51) |
| P_trend_ |  | 0.51 | 0.52 |
| **Rectal**^ǁ^ |  |  |  |
| <0.80 | 87 | 1.00 | 1.00 |
| ≥0.80-≤0.85 | 43 | 1.31 (0.91-1.89) | 1.26 (0.87-1.83) |
| >0.85 | 14 | 0.76 (0.43-1.34) | 0.71 (0.40-1.27) |
| P_trend_ |  | 0.85 | 0.64 |

- All models were adjusted for age at enrollment, education, physical activity, alcohol intake, smoking, height. HR= hazard ratio; CI= confidence interval; WC- waist circumference; WHR- waist to hip ratio
- ^*^Also adjusted for age at menarche, age at first full-term birth and parity combined, HRT status, age at menopause
- ^†^Also adjusted for family history of breast cancer and mammogram ever
- ^ǁ^Also adjusted for diabetes, red meat intake, processed meat intake, fruits and vegetable intake, folate supplement intake, Vitamin D supplement intake
- **^‡^**Also adjusted for BMI

**Supplementary Table 3: Hazard ratios and 95% CI for the association of baseline BIA-derived baseline body fat measures with risk of incident, invasive colorectal cancer among men in the UK Biobank**

| **Body fat measures** | Colorectal  N=357 | |  | Colon  N=199 | |  | Rectal  N=158 | |
| --- | --- | --- | --- | --- | --- | --- | --- | --- |
|  | Cases/incidence per 1000PY | Multivariable-adjusted HR (95% CI) |  | Cases/incidence per 1000PY | Multivariable-adjusted HR (95%  CI) |  | Cases/incidence per  1000PY | Multivariable-adjusted HR (95%  CI) |
| Body fat measures (general) |  |  |  |  |  |  |  |  |
| **FMI (kg/m^2^)** |  |  |  |  |  |  |  |  |
| Q1 | 63/0.71 | 1.00 |  | 32/0.36 | 1.00 |  | 31/0.35 | 1.00 |
| Q2 | 71/0.92 | 1.19 (0.84-1.66) |  | 39/0.51 | 1.30 (0.81-2.08) |  | 32/0.41 | 1.07 (0.65-1.76) |
| Q3 | 77/1.04 | 1.23 (0.88-1.72) |  | 39/0.52 | 1.25 (0.78-2.00) |  | 38/0.51 | 1.21 (0.75-1.95) |
| Q4 | 68/1.09 | 1.18 (0.83-1.66) |  | 44/0.71 | 1.54 (0.97-2.44) |  | 24/0.39 | 0.82 (0.48-1.40) |
| Q5 | 78/1.31 | 1.26 (0.90-1.77) |  | 45/0.76 | 1.49 (0.94-2.37) |  | 33/0.55 | 1.05 (0.63-1.73) |
| P_trend_ ^*^ |  | 0.25 |  |  | 0.07 |  |  | 0.81 |
| Per SD increase |  | 1.03 (0.92-1.14) |  |  | 1.01 (0.86-1.17) |  |  | 1.05 (0.91-1.22) |
| **Body fat %** |  |  |  |  |  |  |  |  |
| Q1 | 57/0.66 | 1.00 |  | 36/0.37 | 1.00 |  | 27/0.31 | 1.00 |
| Q2 | 66/0.83 | 1.12 (0.79-1.60) |  | 31/0.47 | 1.20 (0.74-1.95) |  | 29/0.37 | 1.03 (0.61-1.74) |
| Q3 | 83/1.14 | 1.42 (1.01-2.00) |  | 44/0.66 | 1.45 (0.91-2.31) |  | 39/0.53 | 1.39 (0.85-2.28) |
| Q4 | 69/1.09 | 1.23 (0.86-1.75) |  | 44/0.66 | 1.49 (0.93-2.38) |  | 26/0.41 | 0.94 (0.55-1.63) |
| Q5 | 82/1.37 | 1.35 (0.95-1.91) |  | 44/0.67 | 1.44 (0.90-2.32) |  | 37/0.62 | 1.24 (0.74-2.06) |
| P_trend_ ^*^ |  | 0.09 |  |  | 0.09 |  |  | 0.54 |
| Per SD increase |  | 1.08 (0.97-1.21) |  |  | 1.15 (0.99-1.33) |  |  | 1.01 (0.86-1.19) |
| **Whole body fat mass to whole body fat free mass** |  |  |  |  |  |  |  |  |
| Q1 | 56/0.67 | 1.00 |  | 30/0.36 | 1.00 |  | 26/0.31 | 1.00 |
| Q2 | 66/0.84 | 1.12 (0.78-1.59) |  | 37/0.47 | 1.17 (0.72-1.90) |  | 29/0.37 | 1.05 (0.62-1.78) |
| Q3 | 82/1.12 | 1.39 (0.99-1.96) |  | 43/0.59 | 1.37 (0.86-2.19) |  | 39/0.53 | 1.40 (0.85-2.31) |
| Q4 | 67/1.07 | 1.20 (0.84-1.72) |  | 42/0.67 | 1.43 (0.89-2.30) |  | 25/0.40 | 0.93 (0.54-1.62) |
| Q5 | 86/1.35 | 1.34 (0.95-1.89) |  | 47/0.74 | 1.40 (0.87-2.23) |  | 39/0.61 | 1.27 (0.76-2.11) |
| P_trend_ ^*^ |  | 0.07 |  |  | 0.12 |  |  | 0.52 |
| Per SD increase |  | 1.04 (0.94-1.15) |  |  | 1.01 (0.87-1.17) |  |  | 1.06 (0.92-1.22) |
| **BMI** **(kg/m^2^)** |  |  |  |  |  |  |  |  |
| Q1 | 67/0.88 | 1.00 |  | 41/0.54 | 1.00 |  | 26/0.34 | 1.00 |
| Q2 | 88/1.05 | 1.19 (0.86-1.63) |  | 37/0.44 | 0.82 (0.53-1.28) |  | 51/0.61 | 1.75 (1.09-2.81) |
| Q3 | 64/1.19 | 1.32 (0.93-1.86) |  | 42/0.78 | 1.41 (0.92-2.18) |  | 22/0.41 | 1.16 (0.66-2.06) |
| Q4 | 73/0.86 | 0.92 (0.66-1.28) |  | 35/0.41 | 0.73 (0.47-1.15) |  | 38/0.45 | 1.21 (0.73-1.99) |
| Q5 | 65/1.03 | 1.09 (0.78-1.54) |  | 44/0.69 | 1.23 (0.80-1.88) |  | 21/0.33 | 0.89 (0.50-1.59) |
| P_trend_^*^ |  | 0.75 |  |  | 0.56 |  |  | 0.26 |
| Per SD increase |  | 0.98 (0.89-1.09) |  |  | 1.05 (0.91-1.22) |  |  | 0.91 (0.78-1.06) |
| Body fat measures (central) |  |  |  |  |  |  |  |  |
| **TFMI (kg/m^2^)** |  |  |  |  |  |  |  |  |
| Q1 | 58/0.70 | 1.00 |  | 32/0.37 | 1.00 |  | 27/0.32 | 1.00 |
| Q2 | 71/0.86 | 1.13 (0.80-1.60) |  | 36/0.46 | 1.14 (0.71-1.83) |  | 33/0.40 | 1.12 (0.67-1.86) |
| Q3 | 76/1.02 | 1.23 (0.87-1.74) |  | 41/0.52 | 1.20 (0.75-1.92) |  | 37/0.50 | 1.27 (0.77-2.09) |
| Q4 | 72/1.14 | 1.26 (0.89-1.78) |  | 44/0.71 | 1.51 (0.95-2.39) |  | 27/0.43 | 0.98 (0.57-1.69) |
| Q5 | 80/1.36 | 1.26 (0.89-1.79) |  | 46/0.78 | 1.41 (0.88-2.25) |  | 34/0.58 | 1.12 (0.67-1.88) |
| P_trend_ ^*^ |  | 0.16 |  |  | 0.07 |  |  | 0.88 |
| Per SD increase |  | 1.07 (0.96-1.19) |  |  | 1.14 (0.99-1.32) |  |  | 0.99 (0.84-1.16) |
| **Trunk fat %** |  |  |  |  |  |  |  |  |
| Q1 | 60/0.71 | 1.00 |  | 32/0.38 | 1.00 |  | 28/0.33 | 1.00 |
| Q2 | 68/0.86 | 1.11 (0.78-1.57) |  | 33/0.42 | 1.01 (0.62-1.65) |  | 35/0.44 | 1.21 (0.74-1.99) |
| Q3 | 76/1.02 | 1.24 (0.88-1.74) |  | 46/0.62 | 1.42 (0.90-2.24) |  | 30/0.40 | 1.03 (0.61-1.73) |
| Q4 | 70/1.08 | 1.20 (0.85-1.70) |  | 39/0.60 | 1.28 (0.80-2.04) |  | 31/0.48 | 1.11 (0.66-1.86) |
| Q5 | 83/1.41 | 1.38 (0.98-1.94) |  | 49/0.83 | 1.57 (0.99-2.49) |  | 34/0.58 | 1.16 (0.69-1.94) |
| P_trend_ ^*^ |  | 0.06 |  |  | 0.03 |  |  | 0.74 |
| Per SD increase |  | 1.08 (0.97-1.21) |  |  | 1.15 (0.99-1.33) |  |  | 1.01 (0.86-1.19) |
| **Trunk fat to trunk fat free mass** |  |  |  |  |  |  |  |  |
| Q1 | 58/0.69 | 1.00 |  | 30/0.36 | 1.00 |  | 28/0.34 | 1.00 |
| Q2 | 67/0.86 | 1.14 (0.80-1.62) |  | 35/0.45 | 1.15 (0.71-1.88) |  | 32/0.41 | 1.12 (0.67-1.85) |
| Q3 | 74/1.00 | 1.24 (0.88-1.75) |  | 44/0.60 | 1.44 (0.90-2.30) |  | 30/0.41 | 1.03 (0.61-1.72) |
| Q4 | 75/1.11 | 1.27 (0.90-1.79) |  | 41/0.61 | 1.36 (0.85-2.19) |  | 34/0.50 | 1.16 (0.70-1.92) |
| Q5 | 83/1.39 | 1.38 (0.98-1.95) |  | 49/0.42 | 1.63 (1.02-2.60) |  | 34/0.57 | 1.13 (0.67-1.88) |
| P_trend_ ^*^ |  | 0.06 |  |  | 0.03 |  |  | 0.64 |
| Per SD increase |  | 1.08 (0.97-1.20) |  |  | 1.14 (0.99-1.31) |  |  | 1.01 (0.86-1.19) |
| **Leg fat mass (kg)** |  |  |  |  |  |  |  |  |
| Q1 | 78/0.79 | 1.00 |  | 39/0.40 | 1.00 |  | 39/0.40 | 1.00 |
| Q2 | 82/1.06 | 1.18 (0.86-1.61) |  | 46/0.59 | 1.33 (0.86-2.03) |  | 36/0.46 | 1.03 (0.65-1.62) |
| Q3 | 72/1.10 | 1.15 (0.83-1.59) |  | 36/0.55 | 1.17 (0.74-1.84) |  | 36/0.55 | 1.13 (0.71-1.78) |
| Q4 | 52/0.98 | 0.99 (0.69-1.41) |  | 33/0.62 | 1.27 (0.79-2.02) |  | 19/0.36 | 0.71 (0.41-1.24) |
| Q5 | 73/1.07 | 1.01 (0.73-1.40) |  | 45/0.66 | 1.26 (0.81-1.96) |  | 28/0.41 | 0.76 (0.46-1.26) |
| P_trend_ ^*^ |  | 0.73 |  |  | 0.43 |  |  | 0.16 |
| Per SD increase |  | 0.98 (0.88-1.09) |  |  | 1.05 (0.91-1.20) |  |  | 0.89 (0.75-1.06) |
| **Ratio of trunk fat mass to leg fat mass** |  |  |  |  |  |  |  |  |
| Q1 | 53/0.71 | 1.00 |  | 28/0.38 | 1.00 |  | 25/0.34 | 1.00 |
| Q2 | 67/0.86 | 1.11 (0.77-1.59) |  | 42/0.54 | 1.32 (0.81-2.14) |  | 25/0.32 | 0.87 (0.50-1.52) |
| Q3 | 65/0.77 | 0.92 (0.64-1.32) |  | 34/0.40 | 0.91 (0.55-1.51) |  | 31/0.37 | 0.93 (0.54-1.57) |
| Q4 | 94/1.31 | 1.47 (1.05-2.07) |  | 46/0.64 | 1.36 (0.85-2.18) |  | 48/0.67 | 1.61 (0.98-2.62) |
| Q5 | 78/1.47 | 1.63 (1.14-2.32) |  | 49/0.92 | 1.92 (1.20-3.07) |  | 29/0.55 | 1.30 (0.75-2.23) |
| P_trend_ ^*^ |  | 0.001 |  |  | 0.01 |  |  | 0.04 |
| Per SD increase |  | 1.12 (1.01-1.26) |  |  | 1.16 (1.01-1.33) |  |  | 1.09 (0.92-1.29) |
| **WC (cm)** |  |  |  |  |  |  |  |  |
| Q1 | 79/0.74 | 1.00 |  | 40/0.38 | 1.00 |  | 39/0.37 | 1.00 |
| Q2 | 55/0.85 | 1.05 (0.75-1.49) |  | 31/0.48 | 1.18 (0.74-1.89) |  | 24/0.37 | 0.92 (0.55-1.53) |
| Q3 | 72/1.01 | 1.18 (0.85-1.62) |  | 42/0.59 | 1.36 (0.88-2.11) |  | 30/0.42 | 0.98 (0.61-1.59) |
| Q4 | 85/1.18 | 1.30 (0.95-1.78) |  | 46/0.64 | 1.40 (0.91-2.16) |  | 39/0.54 | 1.19 (0.75-1.88) |
| Q5 | 66/1.41 | 1.40 (0.99-1.97) |  | 40/0.85 | 1.68 (1.05-2.66) |  | 26/0.56 | 1.11 (0.66-1.88) |
| P_trend_^*^ |  | 0.03 |  |  | 0.03 |  |  | 0.44 |
| Per SD increase |  | 1.12 1.01-1.25) |  |  | 1.16 (0.99-1.35) |  |  | 1.07 (0.90-1.27) |
| **WHR** |  |  |  |  |  |  |  |  |
| Q1 | 69/0.74 | 1.00 |  | 36/0.39 | 1.00 |  | 33/0.36 | 1.00 |
| Q2 | 61/0.76 | 0.92 (0.65-1.29) |  | 33/0.41 | 0.96 (0.60-1.54) |  | 28/0.35 | 0.86 (0.52-1.43) |
| Q3 | 79/1.05 | 1.17 (0.85-1.62) |  | 45/0.59 | 1.30 (0.83-2.01) |  | 34/0.45 | 1.03 (0.64-1.67) |
| Q4 | 66/1.13 | 1.19 (0.84-1.67) |  | 39/0.67 | 1.37 (0.87-2.17) |  | 27/0.46 | 0.98 (0.59-1.64) |
| Q5 | 82/1.48 | 1.40 (1.01-1.94) |  | 46/0.83 | 1.55 (0.99-2.43) |  | 36/0.65 | 1.23 (0.76-2.00) |
| P_trend_^*^ |  | 0.01 |  |  | 0.02 |  |  | 0.33 |
| Per SD increase |  | 1.12 (1.02-1.24) |  |  | 1.17 (1.03-1.33) |  |  | 1.06 (0.91-1.24) |

- All models were adjusted for age at enrollment, education, physical activity, alcohol intake, smoking, height, red meat intake, processed meat intake, fruits and vegetable intake, folate supplement intake, Vitamin D supplement intake, history of diabetes. PY= person-year; CI= confidence interval; HR= hazard ratio; SD= standard deviation; WC= waist circumference; WHR= waist to hip ratio
- ^*^All tests were two-sided.
- Ranges: FMI: ≤3.7, 3.8-4.5, 4.6-5.1, 5.2-5.7, >5.7; body fat %: ≤16.4, 16.5-19.2, 19.3-21.4, 21.5-23.7, >23.7; whole body fat to whole body fat free mass: ≤0.20, 0.21-0.24, 0.25-0.27, 0.28-0.31, >0.31; BMI: ≤22.1, 22.2-23.3, 23.4-23.8, 23.9-24.5, >24.5; TFMI: ≤2.22; 2.23-2.81, 2.82-3.25, 3.25-3.68, >3.68; trunk fat %: ≤17.2, 17.3-20.9, 21.0-23.7, 23.8-26.6, >26.6; trunk fat mass to trunk fat free mass: ≤0.20, 0.21-0.26, 0.27-0.31, 0.31-0.36, >0.36; leg fat mass: ≤3.3, 3.4-3.8, 3.9-4.2, 4.3-4.6, >4.6; ratio of trunk fat mass to leg fat mass: ≤1.93, 1.94-2.21, 2.22-2.44, 2.45-2.69, >2.69; WC: ≤82, 83-85, 86-88, 89-92, >92; WHR: ≤0.85, 0.86-0.88, 0.89-0.91, 0.92-0.94, >0.94 for quintiles 1,2 3, 4, and 5, respectively

**Supplementary Table 4: Hazard ratios and 95% CI for the association of baseline BIA-derived baseline body fat measures with risk of incident, invasive colorectal cancer among women in the UK Biobank.**

|  | Colorectum  N=486 | |  | Colon  N= 342 | |  | Rectum  N= 144 | |
| --- | --- | --- | --- | --- | --- | --- | --- | --- |
|  | Cases/incidence per 1000PY | Multivariable-adjusted HR (95% CI) |  | Cases/incidence per  1000PY | Multivariable-adjusted HR (95% CI) |  | Cases/incidence per  1000PY | Multivariable-adjusted HR (95% CI) |
| **FMI (kg/m^2^)** |  |  |  |  |  |  |  |  |
| Q1 | 98/0.60 | 1.00 |  | 72/0.44 | 1.00 |  | 31/0.19 | 1.00 |
| Q2 | 90/0.62 | 0.97 (0.73-1.29) |  | 65/0.45 | 0.94 (0.67-1.32) |  | 26/0.17 | 1.05 (0.60-1.82) |
| Q3 | 101/0.77 | 1.13 (0.85-1.50) |  | 77/0.59 | 1.15 (0.83-1.59) |  | 29/0.21 | 1.07 (0.61 1.87) |
| Q4 | 101/0.79 | 1.10 (0.83-1.46) |  | 62/0.49 | 0.89 (0.63-1.26) |  | 28/0.23 | 1.72 (1.04-2.85) |
| Q5 | 96/0.84 | 1.08 (0.81-1.44) |  | 66/0.58 | 0.98 (0.70-1.38) |  | 30/0.28 | 1.37 (0.80-2.36) |
| P_trend_ ^*^ |  | 0.40 |  |  | 0.81 |  |  | 0.05 |
| Per SD increase |  | 1.01 (0.92-1.10) |  |  | 0.97 (0.86-1.08) |  |  | 1.11 (0.93-1.31) |
| P_heterogeneity_ |  | 0.367 |  |  | 0.070 |  |  | 0.351 |
| **Body fat %** |  |  |  |  |  |  |  |  |
| Q1 | 98/0.62 | 1.00 |  | 68/0.43 | 1.00 |  | 30/0.19 | 1.00 |
| Q2 | 94/0.63 | 0.94 (0.70-1.24) |  | 72/0.48 | 1.02 (0.73-1.42) |  | 22/0.15 | 0.74 (0.43-1.28) |
| Q3 | 105/0.77 | 1.07 (0.81-1.42) |  | 72/0.53 | 1.04 (0.75-1.46) |  | 33/0.24 | 1.16 (0.70-1.91) |
| Q4 | 85/0.70 | 0.92 (0.68-1.23) |  | 59/0.49 | 0.89 (0.62-1.27) |  | 26/0.22 | 0.98 (0.57-1.67) |
| Q5 | 104/0.91 | 1.09 (0.82-1.45) |  | 71/0.62 | 1.04 (0.74-1.47) |  | 33/0.29 | 1.21 (0.72-2.03) |
| P_trend_ ^*^ |  | 0.63 |  |  | 0.90 |  |  | 0.29 |
| Per SD increase |  | 0.99 (0.90-1.08) |  |  | 0.95 (0.85-1.06) |  |  | 1.08 (0.90-1.29) |
| P_heterogeneity_ |  | 0.124 |  |  | 0.093 |  |  | 0.777 |
| **Whole body fat mass to whole body fat free mass** |  |  |  |  |  |  |  |  |
| Q1 | 98/0.62 | 1.00 |  | 68/0.43 | 1.00 |  | 30/0.19 | 1.00 |
| Q2 | 94/0.63 | 0.93 (0.70-1.23) |  | 72/0.48 | 1.01 (0.72-1.41) |  | 22/0.15 | 0.73 (0.42-1.27) |
| Q3 | 104/0.77 | 1.06 (0.80-1.40) |  | 71/0.53 | 1.02 (0.73-1.43) |  | 33/0.24 | 1.15 (0.70-1.90) |
| Q4 | 83/0.70 | 0.90 (0.67-1.22) |  | 58/0.49 | 0.88 (0.62-1.26) |  | 25/0.21 | 0.95 (0.55-1.63) |
| Q5 | 107/0.90 | 1.07 (0.80-1.42) |  | 73/0.61 | 1.02 (0.73-1.44) |  | 34/0.29 | 1.19 (0.71-1.99) |
| P_trend_^*^ |  | 0.71 |  |  | 0.84 |  |  | 0.32 |
| Per SD increase |  | 0.99 (0.91-1.09) |  |  | 0.96 (0.86-1.07) |  |  | 1.09 (0.92-1.29) |
| P_heterogeneity_ |  | 0.126 |  |  | 0.108 |  |  | 0.727 |
| **BMI (kg/m^2^)** |  |  |  |  |  |  |  |  |
| Q1 | 97/0.62 | 1.00 |  | 72/0.46 | 1.00 |  | 25/0.16 | 1.00 |
| Q2 | 96/0.69 | 1.07 (0.80-1.41) |  | 69/0.49 | 1.02 (0.73-1.42) |  | 27/0.19 | 1.20 (0.70-2.07) |
| Q3 | 94/0.70 | 1.06 (0.80-1.41) |  | 63/0.47 | 0.94 (0.67-1.32) |  | 31/0.23 | 1.42 (0.84-2.41) |
| Q4 | 110/0.88 | 1.29 (0.98-1.69) |  | 76/0.61 | 1.17 (0.85-1.62) |  | 34/0.27 | 1.63 (0.97-2.74) |
| Q5 | 89/0.72 | 1.04 (0.78-1.39) |  | 62/0.50 | 0.95 (0.67-1.34) |  | 27/0.22 | 1.30 (0.75-2.24) |
| P_trend_^*^ |  | 0.39 |  |  | 0.90 |  |  | 0.17 |
| Per SD increase |  | 1.03 (0.94-1.13) |  |  | 1.00 (0.90-1.12) |  |  | 1.10 (0.93-1.31) |
| P_heterogeneity_ |  | 0.608 |  |  | 0.621 |  |  | 0.138 |
| **TFMI (kg/m^2^)** |  |  |  |  |  |  |  |  |
| Q1 | 101/0.65 | 1.00 |  | 72/0.45 | 1.00 |  | 29/0.18 | 1.00 |
| Q2 | 95/0.66 | 0.94 (0.71-1.25) |  | 71/0.48 | 0.98 (0.70-1.36) |  | 24/0.16 | 0.86 (0.50-1.47) |
| Q3 | 102/0.77 | 1.06 (0.81-1.40) |  | 69/0.52 | 0.99 (0.71-1.38) |  | 33/0.25 | 1.25 (0.76-2.07) |
| Q4 | 92/0.72 | 0.99 (0.74-1.32) |  | 67/0.54 | 0.98 (0.70-1.37) |  | 25/0.20 | 1.00 (0.58-1.72) |
| Q5 | 96/0.83 | 1.06 (0.80-1.41) |  | 63/0.56 | 0.95 (0.68-1.34) |  | 33/0.29 | 1.37 (0.82-2.27) |
| P_trend_^*^ |  | 0.60 |  |  | 0.82 |  |  | 0.18 |
| Per SD increase |  | 0.99 (0.91-1.09) |  |  | 0.96 (0.86-1.07) |  |  | 1.10 (0.93-1.30) |
| P_heterogeneity_ |  | 0.188 |  |  | 0.067 |  |  | 0.805 |
| **Trunk fat %** |  |  |  |  |  |  |  |  |
| Q1 | 102/0.65 |  |  | 74/0.47 | 1.00 |  | 28/0.18 | 1.00 |
| Q2 | 96/0.61 | 0.87 (0.65-1.15) |  | 69/0.44 | 0.85 (0.61-1.18) |  | 27/0.17 | 0.92 (0.54-1.56) |
| Q3 | 105/0.80 | 1.08 (0.82-1.43) |  | 74/0.57 | 1.03 (0.74-1.44) |  | 31/0.24 | 1.22 (0.72-2.05) |
| Q4 | 89/0.74 | 0.94 (0.70-1.26) |  | 63/0.52 | 0.89 (0.63-1.26) |  | 26/0.21 | 1.06 (0.61-1.85) |
| Q5 | 94/0.84 | 1.01 (0.75-1.36) |  | 62/0.55 | 0.90 (0.63-1.28) |  | 32/0.29 | 1.34 (0.78-2.30) |
| P_trend_^*^ |  | 0.74 |  |  | 0.71 |  |  | 0.23 |
| Per SD increase |  | 0.98 (0.89-1.08) |  |  | 0.94 (0.84-1.06) |  |  | 1.07 (0.89-1.28) |
| P_heterogeneity_ |  | 0.089 |  |  | 0.027 |  |  | 0.915 |
| **Trunk fat to trunk fat free mass** |  |  |  |  |  |  |  |  |
| Q1 | 104/0.68 | 1.00 |  | 74/0.48 | 1.00 |  | 28/0.18 | 1.00 |
| Q2 | 94/0.64 | 0.81 (0.61-1.08) |  | 68/0.43 | 0.82 (0.59-1.14) |  | 24/0.15 | 0.80 (0.46-1.39) |
| Q3 | 118/0.88 | 1.06 (0.81-1.40) |  | 74/0.56 | 0.99 (0.72-1.38) |  | 33/0.25 | 1.25 (0.75-2.08) |
| Q4 | 81/0.62 | 0.90 (0.67-1.22) |  | 61/0.51 | 0.85 (0.60-1.21) |  | 26/0.22 | 1.05 (0.61-1.82) |
| Q5 | 89/0.78 | 1.00 (0.75-1.35) |  | 65/0.57 | 0.89 (0.63-1.27) |  | 33/0.29 | 1.32 (0.77-2.27) |
| P_trend_^*^ |  | 0.72 |  |  | 0.66 |  |  | 0.18 |
| Per SD increase |  | 0.99 (0.90-1.08) |  |  | 0.95 (0.85-1.06) |  |  | 1.08 (0.91-1.28) |
| P_heterogeneity_ |  | 0.080 |  |  | 0.026 |  |  | 0.922 |
| **Leg fat mass (kg)** |  |  |  |  |  |  |  |  |
| Q1 | 112/0.67 | 1.00 |  | 78/0.47 | 1.00 |  | 34/0.20 | 1.00 |
| Q2 | 92/0.62 | 0.89 (0.67-1.17) |  | 69/0.47 | 0.94 (0.68-1.31) |  | 23/0.16 | 0.75 (0.44-1.28) |
| Q3 | 100/0.71 | 0.98 (0.75-1.29) |  | 72/0.51 | 0.99 (0.72-1.38) |  | 28/0.20 | 0.94 (0.57-1.56) |
| Q4 | 91/0.75 | 0.99 (0.75-1.31) |  | 62/0.51 | 0.95 (0.68-1.34) |  | 29/0.24 | 1.10 (0.66-1.82) |
| Q5 | 91/0.90 | 1.16 (0.86-1.56) |  | 61/0.60 | 1.10 (0.77-1.57) |  | 30/0.30 | 1.32 (0.78-2.23) |
| P_trend_^*^ |  | 0.29 |  |  | 0.70 |  |  | 0.18 |
| Per SD increase |  | 1.02 (0.93-1.12) |  |  | 0.98 (0.88-1.10) |  |  | 1.12 (0.94-1.34) |
| P_heterogeneity_ |  | 0.726 |  |  | 0.421 |  |  | 0.140 |
| **Ratio of trunk fat mass to leg fat mass** |  |  |  |  |  |  |  |  |
| Q1 | 104/0.68 | 1.00 |  | 75/0.47 | 1.00 |  | 29/0.19 | 1.00 |
| Q2 | 94/0.64 | 0.90 (0.67-1.19) |  | 63/0.43 | 0.83 (0.59-1.16) |  | 31/0.21 | 1.08 (0.64-1.81) |
| Q3 | 118/0.88 | 1.19 (0.90-1.58) |  | 89/0.55 | 1.24 (0.89-1.72) |  | 29/0.22 | 1.07 (0.62-1.84) |
| Q4 | 81/0.62 | 0.84(0.61-1.16) |  | 56/0.50 | 0.80 (0.55-1.17) |  | 25/0.19 | 0.95 (0.53-1.70) |
| Q5 | 89/0.78 | 1.06 (0.76-1.49) |  | 59/0.60 | 0.97 (0.65-1.46) |  | 30/0.26 | 1.31 (0.71-2.41) |
| P_trend_^*^ |  | 0.88 |  |  | 0.86 |  |  | 0.60 |
| Per SD increase |  | 0.95 (0.85-1.07) |  |  | - 1. (0.81-1.06) |  |  | 1.03 (0.84-1.26) |
| P_heterogeneity_ |  | 0.007 |  |  | 0.014 |  |  | 0.270 |
| **WC (cm)** |  |  |  |  |  |  |  |  |
| Q1 | 104/0.60 | 1.00 |  | 74/0.42 | 1.00 |  | 30/0.17 | 1.00 |
| Q2 | 113/0.66 | 1.06 (0.81-1.39) |  | 76/0.44 | 0.99 (0.72-1.38) |  | 37/0.22 | 1.24 (0.76-2.01) |
| Q3 | 93/0.75 | 1.15 (0.87-1.53) |  | 66/0.53 | 1.14 (0.82-1.59) |  | 27/0.22 | 1.19 (0.70-2.01) |
| Q4 | 88/0.95 | 1.40 (1.05-1.87) |  | 62/0.67 | 1.37 (0.97-1.93) |  | 26/0.28 | 1.49 (0.87-2.54) |
| Q5 | 88/0.75 | 1.08 (0.78-1.40) |  | 64/0.55 | 1.05 (0.74-1.49) |  | 24/0.21 | 1.03 (0.59-1.79) |
| P_trend_^*^ |  | 0.29 |  |  | 0.30 |  |  | 0.69 |
| Per SD increase |  | 1.06 (0.97-1.16) |  |  | 1.05 (0.94-1.17) |  |  | 1.09 (0.92-1.29) |
| P_heterogeneity_ |  | 0.173 |  |  | 0.131 |  |  | 0.632 |
| **WHR** |  |  |  |  |  |  |  |  |
| Q1 | 86/0.56 | 1.00 |  | 59/0.39 | 1.00 |  | 27/0.18 | 1.00 |
| Q2 | 87/0.62 | 1.04 (0.77-1.40) |  | 66/0.47 | 1.15 (0.81-1.64) |  | 21/0.15 | 0.80 (0.45-1.42) |
| Q3 | 107/0.81 | 1.30 (0.98-1.73) |  | 70/0.53 | 1.23 (0.87-1.75) |  | 37/0.28 | 1.46 (0.89-2.40) |
| Q4 | 109/0.84 | 1.28 (0.96-1.70) |  | 75/0.58 | 1.26 (0.90-1.78) |  | 34/0.26 | - 1. 1.32 (0.79-2.19) |
| Q5 | 97/0.78 | 1.12 (0.83-1.51) |  | 72/0.58 | 1.19 (0.84-1.69) |  | 25/0.20 | - 1. 0.98 (0.56-1.70) |
| P_trend_^*^ |  | 0.20 |  |  | 0.28 |  |  | - 1. 0.47 |
| Per SD increase |  | 1.05 (0.96-1.16) |  |  | 1.06 (0.95-1.17) |  |  | - 1. 1.06 (0.90-1.25) |
| P_heterogeneity_ |  | 0.160 |  |  | 0.167 |  |  | 0.523 |

- All models were adjusted for age at enrollment, education, physical activity, alcohol intake, smoking, height, red meat intake, processed meat intake, fruits and vegetable intake, folate supplement intake, Vitamin D supplement intake, history of diabetes. PY= person-year; CI= confidence interval; HR= hazard ratio; SD= standard deviation
- ^*^All tests were two-sided.
- Ranges: FMI: ≤6.0, 6.1-6.9, 7.0-7.6, 7.7-8.4, >8.4; body fat %: ≤27.4, 27.5-30.5, 30.6-32.8, 32.9-35.1, >35.1; whole body fat to whole body fat free mass: ≤0.38, 0.39-0.44, 0.45-0.49, 0.50-0.54,> 0.54; BMI: ≤21.4, 21.5-22.6, 22.7-23.5, 23.6-24.2, >24.2; TFMI: ≤2.86; 2.87-3.44, 3.45-3.90, 3.91-4.39, >4.39; trunk fat %: ≤23.8, 23.9-27.8, 27.9-30.6, 30.7-33.6, >33.6; trunk fat mass to trunk fat free mass: ≤0.31, 0.32-0.38, 0.39-0.44, 0.45-0.50, >0.50; leg fat mass: ≤6.8, 6.9-7.6, 7.7-8.2, 8.3-8.8, >8.8; ratio of trunk fat mass to leg fat mass: ≤1.06, 1.07-1.19, 1.20-1.30, 1.31-1.42, >1.43; WC: ≤70, 71-74, 75-77, 78-80.3, >80.3; WHR: ≤0.74, 0.75-0.77, 0.78-0.80, 0.81-0.83, >0.83 for quintiles 1,2 3, 4, and 5, respectively

**Supplementary Table 5: Hazard ratios and 95% CI for the association of baseline body fat measures with risk of incident, invasive female-specific cancers among women in the UK Biobank in models additionally adjusted for BMI.**

|  | Breast (postmenopausal)^*^  N= 1051 |  | Endometrium  N= 155 |  | Ovary  N=170 |
| --- | --- | --- | --- | --- | --- |
|  | Multivariable-adjusted HR (95% CI) |  | Multivariable-adjusted HR (95% CI) |  | Multivariable-adjusted HR (95% CI) |
| **FMI (kg/m^2^)** |  |  |  |  |  |
| Q1 | 1.00 |  | 1.00 |  | 1.00 |
| Q2 | 1.00 (0.79-1.26) |  | 0.96 (0.55-1.68) |  | 1.27 (0.77-2.10) |
| Q3 | 1.09 (0.84-1.41) |  | 0.81 (0.42-1.57) |  | 0.76 (0.40-1.44) |
| Q4 | 1.13 (0.85-1.50) |  | 1.00 (0.49-2.04) |  | 1.19 (0.61-2.31) |
| Q5 | 1.17 (0.86-1.50) |  | 0.99 (0.45-2.19) |  | 0.82 (0.38-1.80) |
| P_trend_^†^ | 0.21 |  | 0.89 |  | 0.56 |
| Per SD increase | 1.06 (0.94-1.18) |  | 1.08 (0.81-1.45) |  | 0.98 (0.74-1.29) |
| **Body fat %** |  |  |  |  |  |
| Q1 | 1.00 |  | 1.00 |  | 1.00 |
| Q2 | 0.96 (0.77-1.20) |  | 1.12 (0.67-1.88) |  | 0.89 (0.55-1.45) |
| Q3 | 1.09 (0.87-1.37) |  | 0.85 (0.47-1.53) |  | 0.77 (0.45-1.33) |
| Q4 | 1.07 (0.84-1.36) |  | 0.99 (0.54-1.84) |  | 1.15 (0.67-1.98) |
| Q5 | 1.05 (0.82-1.36) |  | 1.19 (0.63-2.26) |  | 0.73 (0.39-1.38) |
| P_trend_^†^ | 0.51 |  | 0.72 |  | 0.71 |
| Per SD increase | 1.04 (0.95-1.14) |  | 1.07 (0.85-1.34) |  | 0.99 (0.80-1.22) |
| **Ratio of whole-body fat mass to whole body fat free mass** |  |  |  |  |  |
| Q1 | 1.00 |  | 1.00 |  | 1.00 |
| Q2 | 0.99 (0.79-1.23) |  | 1.11 (0.66-1.86) |  | 0.92 (0.56-1.49) |
| Q3 | 1.10 (0.87-1.38) |  | 0.78 (0.43-1.42) |  | 0.77 (0.44-1.34) |
| Q4 | 1.08 (0.85-1.37) |  | 1.07 (0.58-1.95) |  | 1.26 (0.74-2.17) |
| Q5 | 1.08 (0.84-1.39) |  | 1.13 (0.59-2.14) |  | 0.73 (0.39-1.38) |
| P_trend_^†^ | 0.44 |  | 0.74 |  | 0.79 |
| Per SD increase | 1.04 (0.96-1.13) |  | 1.05 (0.85-1.31) |  | 0.98 (0.79-1.20) |
| **TFMI (kg/m^2^)** |  |  |  |  |  |
| Q1 | 1.00 |  | 1.00 |  | 1.00 |
| Q2 | 0.98 (0.79-1.23) |  | 1.30 (0.76-2.22) |  | 0.84 (0.51-1.38) |
| Q3 | 1.03 (0.81-1.31) |  | 0.99 (0.53-1.87) |  | 0.76 (0.43-1.34) |
| Q4 | 1.03 (0.80-1.53) |  | 1.10 (0.56-2.15) |  | 1.05 (0.58-1.90) |
| Q5 | 1.16 (0.88-1.54) |  | 1.52 (0.74-3.14) |  | 0.78 (0.39-1.55) |
| P_trend_^†^ | 0.213 |  | 0.42 |  | 0.82 |
| Per SD increase | 1.06 (0.96-1.17) |  | 1.12 (0.87-1.45) |  | 0.97 (0.76-1.23) |
| **Trunk fat %** |  |  |  |  |  |
| Q1 | 1.00 |  | 1.00 |  | 1.00 |
| Q2 | 0.99 (0.80-1.22) |  | 0.86 (0.51-1.43) |  | 0.68 (0.41-1.12) |
| Q3 | 1.08 (0.86-1.35) |  | 0.74 (0.41-1.32) |  | 1.07 (0.65-1.76) |
| Q4 | 1.03 (0.81-1.30) |  | 0.95 (0.53-1.71) |  | 1.17 (0.69-1.99) |
| Q5 | 1.15 (0.90-1.48) |  | 1.18 (0.64-2.18) |  | 0.75 (0.40-1.39) |
| P_trend_^†^ | 0.22 |  | 0.43 |  | 0.91 |
| Per SD increase | 1.05 (0.96-1.14) |  | 1.11 (0.90-1.39) |  | 0.97 (0.79-1.19) |
| **Ratio of trunk fat mass to trunk fat free mass** |  |  |  |  |  |
| Q1 | 1.00 |  | 1.00 |  | 1.00 |
| Q2 | 0.95 (0.83-1.27) |  | 0.86 (0.51-1.45) |  | 0.64 (0.38-1.06) |
| Q3 | 1.08 (0.99-1.51) |  | 0.74 (0.41-1.32) |  | 1.02 (0.62-1.68) |
| Q4 | 1.01 (0.96-1.47) |  | 0.98 (0.55-1.76) |  | 1.15 (0.68-1.95) |
| Q5 | 1.14 (0.89-1.46) |  | 1.21 (0.65-2.22) |  | 0.73 (0.39-1.36) |
| P_trend_^†^ | 0.19 |  | 0.38 |  | 0.92 |
| Per SD increase | 1.05 (0.97-1.15) |  | 1.10 (0.90-1.34) |  | 0.97 (0.79-1.18) |
| **Leg fat mass (kg)** |  |  |  |  |  |
| Q1 | 1.00 |  | 1.00 |  | 1.00 |
| Q2 | 0.99 (0.79-1.25) |  | 0.82 (0.47-1.45) |  | 0.91 (0.54-1.54) |
| Q3 | 0.94 (0.72-1.24) |  | 0.75 (0.38-1.48) |  | 0.91 (0.48-1.72) |
| Q4 | 0.99 (0.73-1.36) |  | 0.75 (0.35-1.64) |  | 0.75 (0.35-1.62) |
| Q5 | 0.90 (0.63-1.30) |  | 0.74 (0.30-1.86) |  | 1.05 (0.44-2.52) |
| P_trend_^†^ | 0.63 |  | 0.62 |  | 0.99 |
| Per SD increase | 1.00 (0.87-1.15) |  | 0.94 (0.66-1.34) |  | 1.03 (0.73-1.45) |
| **Ratio of trunk fat mass to leg fat mass** |  |  |  |  |  |
| Q1 | 1.00 |  | 1.00 |  | 1.00 |
| Q2 | 1.04 (0.85-1.28) |  | 1.14 (0.68-1.90) |  | 0.78 (0.47-1.30) |
| Q3 | 0.97 (0.78-1.21) |  | 0.70 (0.37-1.29) |  | 1.14 (0.70-1.88) |
| Q4 | 0.89 (0.71-1.12) |  | 1.08 (0.61-1.93) |  | 1.16 (0.68-1.97) |
| Q5 | 1.22 (0.96-1.56) |  | 1.31 (0.70-2.46) |  | 1.13 (0.61-2.07) |
| P_trend_^†^ | 0.34 |  | 0.49 |  | 0.36 |
| Per SD increase | 1.05 (0.97-1.14) |  | 1.09 (0.98-1.23) |  | 0.98 (0.80-1.20) |
| **WC (cm)** |  |  |  |  |  |
| Q1 | 1.00 |  | 1.00 |  | 1.00 |
| Q2 | 0.94 (0.77-1.15) |  | 1.38 (0.86-2.22) |  | 0.77 (0.48-1.23) |
| Q3 | 1.03 (0.84-1.28) |  | 0.77 (0.42-1.41) |  | 1.19 (0.73-1.92) |
| Q4 | 1.01 (0.80-1.27) |  | 0.95 (0.50-1.79) |  | 0.92 (0.51-1.62) |
| Q5 | 1.07 (0.85-1.34) |  | 1.41 (0.78-2.53) |  | 0.91 (0.51-1.59) |
| P_trend_^†^ | 0.386 |  | 0.64 |  | 0.99 |
| Per SD increase | 1.07 (0.99-1.15) |  | 1.14 (0.94-1.39) |  | 0.97 (0.80-1.18) |
| **WHR** |  |  |  |  |  |
| Q1 | 1.00 |  | 1.00 |  | 1.00 |
| Q2 | 1.06 (0.87-1.29) |  | 0.82 (0.50-1.36) |  | 1.47 (0.94-2.31) |
| Q3 | 1.10 (0.91-1.35) |  | 1.16 (0.73-1.85) |  | 0.83 (0.49-1.40) |
| Q4 | 1.04 (0.85-1.27) |  | 0.73 (0.43-1.24) |  | 1.30 (0.81-2.07) |
| Q5 | 1.10 (0.91-1.35) |  | 1.10 (0.67-1.80) |  | 0.79 (0.46-1.35) |
| P_trend_^†^ | 0.43 |  | 0.89 |  | 0.35 |
| Per SD increase | 1.03 (0.97-1.10) |  | 1.12 (0.95-1.32) |  | 0.93 (0.79-1.09) |

- All models were adjusted for age at enrollment, education, age at menarche, age at first full-term birth and parity combined, HRT status, age at menopause, physical activity, height, alcohol intake, smoking, BMI. CI= confidence interval; HR= hazard ratio; SD= standard deviation; FMI= fat mass index; TFMI= trunk fat mass index; WC= waist circumference; WHR= waist to hip ratio
- ^*^Also adjusted for family history of breast cancer and mammogram ever
- ^†^All tests were two-sided.
- Ranges: FMI: ≤6.0, 6.1-6.9, 7.0-7.6, 7.7-8.4, >8.4; body fat %: ≤27.4, 27.5-30.5, 30.6-32.8, 32.9-35.1, >35.1; whole body fat to whole body fat free mass: ≤0.38, 0.39-0.44, 0.45-0.49, 0.50-0.54,> 0.54; TFMI: ≤2.86; 2.87-3.44, 3.45-3.90, 3.91-4.39, >4.39; trunk fat %: ≤23.8, 23.9-27.8, 27.9-30.6, 30.7-33.6, >33.6; trunk fat mass to trunk fat free mass: ≤0.31, 0.32-0.38, 0.39-0.44, 0.45-0.50, >0.50; leg fat mass: ≤6.8, 6.9-7.6, 7.7-8.2, 8.3-8.8, >8.8; ratio of trunk fat mass to leg fat mass: ≤1.06, 1.07-1.19, 1.20-1.30, 1.31-1.42, >1.43; WC: ≤70, 71-74, 75-77, 78-80.3, >80.3; WHR: ≤0.74, 0.75-0.77, 0.78-0.80, 0.81-0.83, >0.83 for quintiles 1,2 3, 4, and 5, respectively

**Supplementary Table 6: Hazard ratios and 95% CI for the association of baseline BIA-derived baseline body fat measures with risk of incident, invasive colorectal cancer among men and women (combined) in the UK Biobank in models additionally adjusted for BMI.**

| **Body fat measures** | Colorectal  N= 843 | Colon  N= 541 | Rectal  N= 302 |
| --- | --- | --- | --- |
|  | Multivariable-adjusted HR (95% CI) | Multivariable-adjusted HR (95%  CI) | Multivariable-adjusted HR (95%  CI) |
| **FMI (kg/m^2^)** |  |  |  |
| Q1 | 1.00 | 1.00 | 1.00 |
| Q2 | 1.10 (0.87-1.39) | 1.07 (0.79-1.43) | 1.16 (0.78-1.72) |
| Q3 | 1.25 (0.97-1.61) | 1.21 (0.88-1.66) | 1.33 (0.87-2.03) |
| Q4 | 1.24 (0.94-1.63) | 1.12 (0.79-1.59) | 1.46 (0.93-2.29) |
| Q5 | 1.27 (0.95-1.71) | 1.17 (0.80-1.70) | 1.47 (0.91-2.38) |
| P_trend_ ^*^ | 0.11 | 0.48 | 0.09 |
| **Body fat %** |  |  |  |
| Q1 | 1.00 | 1.00 | 1.00 |
| Q2 | 1.02 (0.81-1.28) | 1.07 (0.81-1.43) | 0.91 (0.62-1.35) |
| Q3 | 1.22 (0.97-1.55) | 1.16 (0.87-1.56) | 1.35 (0.92-1.97) |
| Q4 | 1.06 (0.82-1.36) | 1.07 (0.78-1.46) | 1.04 (0.68-1.59) |
| Q5 | 1.22 (0.94-1.58) | 1.15 (0.83-1.59) | 1.35 (0.89-2.05) |
| P_trend_ ^*^ | 0.18 | 0.51 | 0.13 |
| **Whole body fat mass to whole body fat free mass** |  |  |  |
| Q1 | 1.00 | 1.00 | 1.00 |
| Q2 | 1.00 (0.80-1.26) | 1.05 (0.79-1.40) | 0.91 (0.62-1.35) |
| Q3 | 1.20 (0.95-1.51) | 1.12 (0.83-1.50) | 1.34 (0.92-1.96) |
| Q4 | 1.03 (0.80-1.33) | 1.04 (0.76-1.42) | 1.01 (0.66-1.54) |
| Q5 | 1.19 (0.92-1.53) | 1.11 (0.80-1.53) | 1.33 (0.88-2.02) |
| P_trend_ ^*^ | 0.22 | 0.64 | 0.14 |
| **TFMI (kg/m^2^)** |  |  |  |
| Q1 | 1.00 | 1.00 | 1.00 |
| Q2 | 1.03 (0.82-1.29) | 1.02 (0.77-1.36) | 1.04 (0.71-1.53) |
| Q3 | 1.16 (0.91-1.48) | 1.05 (0.77-1.42) | 1.37 (0.92-2.04) |
| Q4 | 1.13 (0.87-1.47) | 1.13 (0.82-1.57) | 1.11 (0.71-1.73) |
| Q5 | 1.19 (0.91-1.58) | 1.08 (0.76-1.53) | 1.41 (0.90-2.23) |
| P_trend_ ^*^ | 0.17 | 0.52 | 0.16 |
| **Trunk fat %** |  |  |  |
| Q1 | 1.00 | 1.00 | 1.00 |
| Q2 | 0.96 (0.76-1.20) | 0.89 (0.67-1.17) | 1.09 (0.75-1.58) |
| Q3 | 1.15 (0.92-1.44) | 1.14 (0.86-1.50) | 1.17 (0.79-1.72) |
| Q4 | 1.04 (0.82-1.33) | 0.98 (0.73-1.33) | 1.15 (0.77-1.72) |
| Q5 | 1.16(0.90-1.48) | 1.06 (0.78-1.45) | 1.34 (0.89-2.02) |
| P_trend_ ^*^ | 0.18 | 0.51 | 0.18 |
| **Trunk fat to trunk fat free mass** |  |  |  |
| Q1 | 1.00 | 1.00 | 1.00 |
| Q2 | 0.93 (0.74-1.17) | 0.90 (0.68-1.20) | 0.98 (0.67-1.43) |
| Q3 | 1.14 (0.90-1.43) | 1.10 (0.83-1.47) | 1.19 (0.81-1.74) |
| Q4 | 1.04 (0.81-1.33) | 0.97 (0.72-1.32) | 1.18 (0.79-1.75) |
| Q5 | 1.15 (0.90-1.48) | 1.06 (0.78-1.45) | 1.32 (0.87-1.98) |
| P_trend_ ^*^ | 0.16 | 0.56 | 0.12 |
| **Leg fat mass (kg)** |  |  |  |
| Q1 | 1.00 | 1.00 | 1.00 |
| Q2 | 1.01 (0.80-1.28) | 1.10 (0.82-1.47) | 0.89 (0.60-1.31) |
| Q3 | 1.06 (0.81-1.38) | 1.09 (0.77-1.53) | 1.02 (0.66-1.57) |
| Q4 | 1.00 (0.74-1.36) | 1.09 (0.74-1.60) | 0.88 (0.53-1.44) |
| Q5 | 1.10 (0.79-1.53) | 1.19 (0.78-1.82) | 0.97 (0.57-1.64) |
| P_trend_ ^*^ | 0.65 | 0.53 | 0.95 |
| **Ratio of trunk fat mass to leg fat mass** |  |  |  |
| Q1 | 1.00 | 1.00 | 1.00 |
| Q2 | 0.97 (0.77-1.20) | 0.95 (0.72-1.26) | 0.98 (0.67-1.44) |
| Q3 | 1.06 (0.85-1.32) | 1.09 (0.83-1.43) | 1.01 (0.69-1.47) |
| Q4 | 1.08 (0.86-1.35) | 0.95 (0.71-1.26) | 1.33 (0.92-1.92) |
| Q5 | 1.25 (0.99-1.59) | 1.21 (0.90-1.62) | 1.33 (0.90-1.97) |
| P_trend_ ^*^ | 0.04 | 0.29 | 0.04 |
| **WC (cm)** |  |  |  |
| Q1 | 1.00 | 1.00 | 1.00 |
| Q2 | 1.11 (0.89-1.38) | 1.11 (0.84-1.46) | 1.12 (0.78-1.61) |
| Q3 | 1.23 (0.98-1.55) | 1.29 (0.97-1.73) | 1.13 (0.77-1.66) |
| Q4 | 1.45 (1.13-1.84) | 1.47 (1.08-2.00) | 1.40 (0.94-2.08) |
| Q5 | 1.27 (0.98-1.66) | 1.35 (0.97-1.88) | 1.14 (0.73-1.78) |
| P_trend_^*^ | 0.02 | 0.02 | 0.33 |
| **WHR** |  |  |  |
| Q1 | 1.00 | 1.00 | 1.00 |
| Q2 | 0.99 (0.80-1.25) | 1.09 (0.82-1.44) | 0.86 (0.59-1.25) |
| Q3 | 1.25 (1.01-1.55) | 1.25 (0.95-1.65) | 1.24 (0.87-1.76) |
| Q4 | 1.25 (1.00-1.56) | 1.30 (0.99-1.71) | 1.17 (0.81-1.69) |
| Q5 | 1.25 (0.99-1.57) | 1.31 (0.99-1.72) | 1.14 (0.78-1.67) |
| P_trend_^*^ | 0.011 | 0.03 | 0.20 |

- All models were adjusted for age at enrollment, education, physical activity, alcohol intake, smoking, height, red meat intake, processed meat intake, fruits and vegetable intake, folate supplement intake, Vitamin D supplement intake, history of diabetes, BMI. CI= confidence interval; HR= hazard ratio; SD= standard deviation; FMI= fat mass index; TFMI= trunk fat mass index; WC= waist circumference; WHR= waist to hip ratio
- ^*^All tests were two-sided.
- Ranges:
- Men: FMI: ≤3.7, 3.8-4.5, 4.6-5.1, 5.2-5.7, >5.7; body fat %: ≤16.4, 16.5-19.2, 19.3-21.4, 21.5-23.7, >23.7; whole body fat to whole body fat free mass: ≤0.20, 0.21-0.24, 0.25-0.27, 0.28-0.31, >0.31; TFMI: ≤2.22; 2.23-2.81, 2.82-3.25, 3.25-3.68, >3.68; trunk fat %: ≤17.2, 17.3-20.9, 21.0-23.7, 23.8-26.6, >26.6; trunk fat mass to trunk fat free mass: ≤0.20, 0.21-0.26, 0.27-0.31, 0.31-0.36, >0.36; leg fat mass: ≤3.3, 3.4-3.8, 3.9-4.2, 4.3-4.6, >4.6; ratio of trunk fat mass to leg fat mass: ≤1.93, 1.94-2.21, 2.22-2.44, 2.45-2.69, >2.69; WC: ≤82, 83-85, 86-88, 89-92, >92; WHR: ≤0.85, 0.86-0.88, 0.89-0.91, 0.92-0.94, >0.94 for quintiles 1,2 3, 4, and 5, respectively
- Women: FMI: ≤6.0, 6.1-6.9, 7.0-7.6, 7.7-8.4, >8.4; body fat %: ≤27.4, 27.5-30.5, 30.6-32.8, 32.9-35.1, >35.1; whole body fat to whole body fat free mass: ≤0.38, 0.39-0.44, 0.45-0.49, 0.50-0.54,> 0.54; TFMI: ≤2.86; 2.87-3.44, 3.45-3.90, 3.91-4.39, >4.39; trunk fat %: ≤23.8, 23.9-27.8, 27.9-30.6, 30.7-33.6, >33.6; trunk fat mass to trunk fat free mass: ≤0.31, 0.32-0.38, 0.39-0.44, 0.45-0.50, >0.50; leg fat mass: ≤6.8, 6.9-7.6, 7.7-8.2, 8.3-8.8, >8.8; ratio of trunk fat mass to leg fat mass: ≤1.06, 1.07-1.19, 1.20-1.30, 1.31-1.42, >1.43; WC: ≤70, 71-74, 75-77, 78-80.3, >80.3; WHR: ≤0.74, 0.75-0.77, 0.78-0.80, 0.81-0.83, >0.83 for quintiles 1,2 3, 4, and 5, respectively

**Supplementary Table 7: Hazard ratios and 95% CI for the association of baseline BIA-derived baseline body fat measures with risk of incident, invasive colorectal cancer among men and women in the UK Biobank in models additionally adjusted for BMI.**

|  | Males | | |  | Females | | |
| --- | --- | --- | --- | --- | --- | --- | --- |
| **Body fat measures** | Colorectal  N= 357 | Colon  N= 199 | Rectal  N= 158 |  | Colorectal  N= 486 | Colon  N= 342 | Rectal  N= 144 |
|  | Multivariable-adjusted HR (95% CI) | | |  | Multivariable-adjusted HR (95% CI) | | |
| **FMI (kg/m^2^)** |  |  |  |  |  |  |  |
| Q1 | 1.00 | 1.00 | 1.00 |  | 1.00 | 1.00 | 1.00 |
| Q2 | 1.28 (0.90-1.82) | 1.35 (0.83-2.20) | 1.20 (0.71-2.01) |  | 0.97 (0.71-1.34) | 0.92 (0.63-1.34) | 1.13 (0.61-2.10) |
| Q3 | 1.38 (0.95-2.00) | 1.32 (0.79-2.21) | 1.44 (0.84-2.45) |  | 1.13 (0.79-1.63) | 1.10 (0.72-1.68) | 1.21 (0.60-2.46) |
| Q4 | 1.35 (0.91-2.01) | 1.65 (0.97-2.80) | 1.01 (0.55-1.86) |  | 1.11 (0.74-1.66) | 0.85 (0.52-1.37) | 2.02 (0.95-4.30) |
| Q5 | 1.48 (0.99-2.21) | 1.60 (0.93-2.77) | 1.33 (0.73-2.42) |  | 1.09 (0.69-1.72) | 0.92 (0.54-1.57) | 1.67 (0.70-3.97) |
| P_trend_ ^*^ | 0.10 | 0.09 | 0.59 |  | 0.62 | 0.65 | 0.11 |
| Per SD increase | 1.03 (0.93-1.14) | 1.00 (0.85-1.18) | 1.05 (0.92-1.20) |  | 0.94 (0.80-1.11) | 0.90 (0.74-1.09) | 1.04 (0.77-1.41) |
| **Body fat %** |  |  |  |  |  |  |  |
| Q1 | 1.00 | 1.00 | 1.00 |  | 1.00 | 1.00 | 1.00 |
| Q2 | 1.18 (0.82-1.70) | 1.21 (0.74-1.98) | 1.14 (0.67-1.95) |  | 0.91 (0.68-1.23) | 1.01 (0.71-1.44) | 0.69 (0.39-1.23) |
| Q3 | 1.54 (1.07-2.20) | 1.47 (0.90-2.40) | 1.62 (0.96-2.73) |  | 1.03 (0.75-1.41) | 1.03 (0.71-1.50) | 1.04 (0.59-1.83) |
| Q4 | 1.35 (0.92-1.97) | 1.51 (0.91-2.50) | 1.14 (0.63-2.03) |  | 0.87 (0.62-1.23) | 0.88 (0.58-1.33) | 0.85 (0.45-1.60) |
| Q5 | 1.50 (1.02-2.20) | 1.47 (0.87-2.47) | 1.53 (0.87-2.70) |  | 1.02 (0.71-1.47) | 1.02 (0.66-1.58) | 1.03 (0.53-1.98) |
| P_trend_ ^*^ | 0.04 | 0.12 | 0.20 |  | 0.95 | 0.85 | 0.67 |
| Per SD increase | 1.12 (0.99-1.27) | 1.16 (0.98-1.36) | 1.07 (0.89-1.29) |  | 0.94 (0.83-1.06) | 0.91 (0.79-1.06) | 1.01 (0.80-1.28) |
| **Whole body fat mass to whole body fat free mass** |  |  |  |  |  |  |  |
| Q1 | 1.00 | 1.00 | 1.00 |  | 1.00 | 1.00 | 1.00 |
| Q2 | 1.16 (0.81-1.68) | 1.17 (0.72-1.92) | 1.15 (0.67-1.98) |  | 0.90 (0.67-1.21) | 1.00 (0.70-1.42) | 0.68 (0.38-1.21) |
| Q3 | 1.48 (1.04-2.12) | 1.37 (0.84-2.24) | 1.62 (0.96-2.74) |  | 1.01 (0.74-1.39) | 1.01 (0.69-1.47) | 1.03 (0.58-1.81) |
| Q4 | 1.39 (0.88-1.90) | 1.43 (0.86-2.38) | 1.11 (0.61-1.99) |  | 0.85 (0.60-1.20) | 0.86 (0.57-1.31) | 0.82 (0.44-1.54) |
| Q5 | 1.46 (1.00-2.12) | 1.40 (0.84-2.32) | 1.53 (0.88-2.66) |  | 0.99 (0.69-1.43) | 0.99 (0.65-1.53) | 0.99 (0.52-1.92) |
| P_trend_ ^*^ | 0.06 | 0.16 | 0.21 |  | 0.95 | 0.77 | 0.73 |
| Per SD increase | 1.03 (0.93-1.14) | 1.01 (0.86-1.18) | 1.05 (0.92-1.20) |  | 0.96 (0.85-1.08) | 0.93 (0.80-1.07) | 1.04 (0.83-1.30) |
| **TFMI (kg/m^2^)** |  |  |  |  |  |  |  |
| Q1 | 1.00 | 1.00 | 1.00 |  | 1.00 | 1.00 | 1.00 |
| Q2 | 1.21 (0.84-1.73) | 1.16 (0.71-1.90) | 1.25 (0.74-2.12) |  | 0.92 (0.68-1.25) | 0.96 (0.68-1.37) | 0.81 (0.46-1.45) |
| Q3 | 1.36 (0.94-1.98) | 1.23 (0.74-2.06) | 1.52 (0.88-2.61) |  | 1.02 (0.73-1.42) | 0.97 (0.65-1.44) | 1.16 (0.63-2.11) |
| Q4 | 1.42 (0.96-2.10) | 1.56 (0.93-2.62) | 1.22 (0.67-2.22) |  | 0.94 (0.65-1.36) | 0.96 (0.62-1.48) | 0.90 (0.45-1.80) |
| Q5 | 1.47 (0.98-2.21) | 1.47 (0.85-2.55) | 1.46 (0.79-2.69) |  | 1.00 (0.67-1.51) | 0.92 (0.57-1.50) | 1.20 (0.57-2.52) |
| P_trend_ ^*^ | 0.06 | 0.09 | 0.34 |  | 0.92 | 0.78 | 0.54 |
| Per SD increase | 1.12 (0.98-1.28) | 1.16 (0.97-1.39) | 1.07 (0.88-1.30) |  | 0.95 (0.84-1.09) | 0.92 (0.79-1.07) | 1.05 (0.83-1.33) |
| **Trunk fat %** |  |  |  |  |  |  |  |
| Q1 | 1.00 | 1.00 | 1.00 |  | 1.00 | 1.00 | 1.00 |
| Q2 | 1.16 (0.81-1.65) | 1.02 (0.62-1.68) | 1.32 (0.79-2.19) |  | 0.84 (0.63-1.13) | 0.83 (0.59-1.17) | 0.87 (0.50-1.51) |
| Q3 | 1.32 (0.92-1.88) | 1.44 (0.90-2.31) | 1.16 (0.68-1.99) |  | 1.04 (0.77-1.41) | 1.01 (0.70-1.44) | 1.13 (0.64-1.99) |
| Q4 | 1.29 (0.89-1.87) | 1.30 (0.79-2.14) | 1.28 (0.74-2.21) |  | 0.89 (0.64-1.24) | 0.86 (0.58-1.28) | 0.97 (0.52-1.80) |
| Q5 | 1.51 (1.04-2.19) | 1.60 (0.98-2.64) | 1.38 (0.79-2.42) |  | 0.95 (0.66-1.35) | 0.86 (0.56-1.32) | 1.18 (0.62-2.27) |
| P_trend_ ^*^ | 0.03 | 0.04 | 0.36 |  | 0.95 | 0.63 | 0.52 |
| Per SD increase | 1.12 (0.98-1.26) | 1.15 (0.98-1.36) | 1.07 (0.89-1.28) |  | 0.94 (0.83-1.06) | 0.91 (0.79-1.05) | 0.99 (0.89-1.25) |
| **Trunk fat to trunk fat free mass** |  |  |  |  |  |  |  |
| Q1 | 1.00 | 1.00 | 1.00 |  | 1.00 | 1.00 | 1.00 |
| Q2 | 1.19 (0.83-1.70) | 1.17 (0.71-1.92) | 1.21 (0.72-2.04) |  | 0.79 (0.59-1.06) | 0.80 (0.57-1.13) | 0.77 (0.43-1.35) |
| Q3 | 1.32 (0.92-1.90) | 1.46 (0.90-2.38) | 1.16 (0.68-1.99) |  | 1.02 (0.75-1.37) | 0.96 (0.67-1.38) | 1.16 (0.66-2.04) |
| Q4 | 1.37 (0.95-1.98) | 1.39 (0.84-2.29) | 1.34 (0.78-2.30) |  | 0.86 (0.61-1.19) | 0.82 (0.55-1.21) | 0.96 (0.52-1.79) |
| Q5 | 1.52 (1.04-2.21) | 1.67 (1.01-2.77) | 1.34 (0.77-2.35) |  | 0.94 (0.66-1.33) | 0.85 (0.55-1.29) | 1.18 (0.62-2.27) |
| P_trend_ ^*^ | 0.03 | 0.04 | 0.29 |  | 0.97 | 0.57 | 0.42 |
| Per SD increase | 1.11 (0.99-1.25) | 1.14 (0.98-1.33) | 1.07 (0.90-1.27) |  | 0.95 (0.84-1.07) | 0.92 (0.80-1.06) | 1.02 (0.82-1.27) |
| **Leg fat mass (kg)** |  |  |  |  |  |  |  |
| Q1 | 1.00 | 1.00 | 1.00 |  | 1.00 | 1.00 | 1.00 |
| Q2 | 1.23 (0.87-1.74) | 1.33 (0.82-2.14) | 1.12 (0.67-1.87) |  | 0.89 (0.64-1.23) | 0.98 (0.67-1.44) | 0.69 (0.37-1.27) |
| Q3 | 1.22 (0.82-1.79) | 1.17 (0.68-2.01) | 1.27 (0.73-2.22) |  | 0.98 (0.67-1.44) | 1.06 (0.67-1.68) | 0.82 (0.40-1.67) |
| Q4 | 1.06 (0.68-1.64) | 1.27 (0.71-2.29) | 0.82 (0.42-1.60) |  | 0.99 (0.63-1.56) | 1.03 (0.60-1.76) | 0.92 (0.41-2.08) |
| Q5 | 1.09 (0.70-1.70) | 1.26 (0.69-2.30) | 0.90 (0.46-1.75) |  | 1.16 (0.69-1.96) | 1.21 (0.64-2.27) | 1.06 (0.41-2.74) |
| P_trend_ ^*^ | 0.84 | 0.69 | 0.44 |  | 0.38 | 0.53 | 0.52 |
| Per SD increase | 0.98 (0.84-1.15) | 1.02 (0.84-1.24) | 0.93 (0.73-1.19) |  | 0.96 (0.78-1.17) | 0.91 (0.72-1.16) | 1.08 (0.74-1.58) |
| **Ratio of trunk fat mass to leg fat mass** |  |  |  |  |  |  |  |
| Q1 | 1.00 | 1.00 | 1.00 |  | 1.00 | 1.00 | 1.00 |
| Q2 | 1.11 (0.77-1.60) | 1.29 (0.79-2.09) | 0.91 (0.52-1.59) |  | 0.88 (0.66-1.18) | 0.82 (0.58-1.17) | 1.02 (0.60-1.72) |
| Q3 | 0.92 (0.64-1.33) | 0.88 (0.53-1.47) | 0.97 (0.57-1.67) |  | 1.16 (0.87-1.56) | 1.23 (0.88-1.74) | 0.98 (0.56-1.73) |
| Q4 | 1.48 (1.05-2.09) | 1.32 (0.82-2.13) | 1.68 (1.02-2.75) |  | 0.81 (0.58-1.13) | 0.79 (0.53-1.18) | 0.86 (0.46-1.58) |
| Q5 | 1.63 (1.15-2.33) | 1.90 (1.19-3.04) | 1.31 (0.76-2.26) |  | 1.02 (0.71-1.46) | 0.96 (0.62-1.48) | 1.15 (0.60-2.22) |
| P_trend_ ^*^ | 0.001 | 0.001 | 0.03 |  | 0.92 | 0.83 | 0.90 |
| Per SD increase | 1.13 (1.01-1.26) | 1.16 (1.01-1.33) | 1.09 (0.93-1.29) |  | 0.93 (0.82-1.05) | 0.91 (0.79-1.05) | 0.97 (0.78-1.22) |
| **WC (cm)** |  |  |  |  |  |  |  |
| Q1 | 1.00 | 1.00 | 1.00 |  | 1.00 | 1.00 | 1.00 |
| Q2 | 1.16 (0.81-1.66) | 1.24 (0.76-2.02) | 1.07 (0.63-1.83) |  | 1.07 (0.81-1.41) | 1.04 (0.74-1.45) | 1.14 (0.69-1.90) |
| Q3 | 1.34 (0.94-1.91) | 1.46 (0.90-2.35) | 1.21 (0.72-2.06) |  | 1.16 (0.85-1.58) | 1.20 (0.83-1.74) | 1.06 (0.59-1.88) |
| Q4 | 1.54 (1.07-2.21) | 1.52 (0.92-2.51) | 1.56 (0.91-2.65) |  | 1.41 (1.01-1.95) | 1.47 (0.99-2.17) | 1.29 (0.71-2.37) |
| Q5 | 1.72 (1.14-2.59) | 1.86 (1.07-3.24) | 1.54 (0.83-2.87) |  | 1.05 (0.74-1.48) | 1.14 (0.75-1.71) | 0.87 (0.45-1.66) |
| P_trend_^*^ | 0.01 | 0.03 | 0.08 |  | 0.42 | 0.23 | 0.75 |
| Per SD increase | 1.21 (1.06-1.40) | 1.20 (0.99-1.45) | 1.23 (0.99-1.52) |  | 1.06 (0.95-1.19) | 1.07 (0.94-1.23) | 1.04 (0.85-1.28) |
| **WHR** |  |  |  |  |  |  |  |
| Q1 | 1.00 | 1.00 | 1.00 |  | 1.00 | 1.00 | 1.00 |
| Q2 | 0.94 (0.67-1.34) | 0.96 (0.60-1.55) | 9.92 (0.55-1.54) |  | 1.04 (0.77-1.40) | 1.16 (0.81-1.64) | 0.79 (0.45-1.40) |
| Q3 | 1.22 (0.88-1.70) | 1.30 (0.83-2.04) | 1.13 (0.69-1.86) |  | 1.29 (0.97-1.72) | 1.24 (0.87-1.75) | 1.42 (0.86-2.34) |
| Q4 | 1.25 (0.88-1.78) | 1.37 (0.85-2.21) | 1.10 (0.65-1.88) |  | 1.26 (0.94-1.68) | 1.27 (0.90-1.80) | 1.25 (0.75-2.10) |
| Q5 | 1.49 (1.05-2.11) | 1.56 (0.97-2.49) | 1.41 (0.85-2.35) |  | 1.10 (0.82-1.49) | 1.20 (0.84-1.72) | 0.91 (0.52-1.60) |
| P_trend_^*^ | 0.01 | 0.02 | 0.14 |  | 0.26 | 0.27 | 0.66 |
| Per SD increase | 1.12 (1.02-1.24) | 1.17 (1.03-1.33) | 1.06 (0.91-1.24) |  | 1.05 (0.96-1.15) | 1.06 (0.95-1.18) | 1.03 (0.87-1.23) |

- All models were adjusted for age at enrollment, education, physical activity, alcohol intake, smoking, height, red meat intake, processed meat intake, fruits and vegetable intake, folate supplement intake, Vitamin D supplement intake, history of diabetes, BMI. CI= confidence interval; HR= hazard ratio; SD= standard deviation; FMI= fat mass index; TFMI= trunk fat mass index; WC= waist circumference; WHR= waist to hip ratio
- ^*^All tests were two-sided.
- Ranges:
- Men: FMI: ≤3.7, 3.8-4.5, 4.6-5.1, 5.2-5.7, >5.7; body fat %: ≤16.4, 16.5-19.2, 19.3-21.4, 21.5-23.7, >23.7; whole body fat to whole body fat free mass: ≤0.20, 0.21-0.24, 0.25-0.27, 0.28-0.31, >0.31; TFMI: ≤2.22; 2.23-2.81, 2.82-3.25, 3.25-3.68, >3.68; trunk fat %: ≤17.2, 17.3-20.9, 21.0-23.7, 23.8-26.6, >26.6; trunk fat mass to trunk fat free mass: ≤0.20, 0.21-0.26, 0.27-0.31, 0.31-0.36, >0.36; leg fat mass: ≤3.3, 3.4-3.8, 3.9-4.2, 4.3-4.6, >4.6; ratio of trunk fat mass to leg fat mass: ≤1.93, 1.94-2.21, 2.22-2.44, 2.45-2.69, >2.69; WC: ≤82, 83-85, 86-88, 89-92, >92; WHR: ≤0.85, 0.86-0.88, 0.89-0.91, 0.92-0.94, >0.94 for quintiles 1,2 3, 4, and 5, respectively
- Women: FMI: ≤6.0, 6.1-6.9, 7.0-7.6, 7.7-8.4, >8.4; body fat %: ≤27.4, 27.5-30.5, 30.6-32.8, 32.9-35.1, >35.1; whole body fat to whole body fat free mass: ≤0.38, 0.39-0.44, 0.45-0.49, 0.50-0.54,> 0.54; TFMI: ≤2.86; 2.87-3.44, 3.45-3.90, 3.91-4.39, >4.39; trunk fat %: ≤23.8, 23.9-27.8, 27.9-30.6, 30.7-33.6, >33.6; trunk fat mass to trunk fat free mass: ≤0.31, 0.32-0.38, 0.39-0.44, 0.45-0.50, >0.50; leg fat mass: ≤6.8, 6.9-7.6, 7.7-8.2, 8.3-8.8, >8.8; ratio of trunk fat mass to leg fat mass: ≤1.06, 1.07-1.19, 1.20-1.30, 1.31-1.42, >1.43; WC: ≤70, 71-74, 75-77, 78-80.3, >80.3; WHR: ≤0.74, 0.75-0.77, 0.78-0.80, 0.81-0.83, >0.83 for quintiles 1,2 3, 4, and 5, respectively

**Supplementary Table 8: Hazard ratios and 95% CI for the association of baseline BIA-derived baseline body fat measures with risk of incident, invasive colorectal cancer among men and women in the UK Biobank (excluding participants within two years of recruitment)**

|  | Males | | |  | Females | | |
| --- | --- | --- | --- | --- | --- | --- | --- |
| **Body fat measures** | Colorectal | Colon | Rectal |  | Colorectal | Colon | Rectal |
|  | Multivariable-adjusted HR (95% CI) | | |  | Multivariable-adjusted HR (95% CI) | | |
| **FMI (kg/m^2^)** |  |  |  |  |  |  |  |
| Q1 | 1.00 | 1.00 | 1.00 |  | 1.00 | 1.00 | 1.00 |
| Q2 | 1.12 (0.76-1.66) | 1.38 (0.81-2.36) | 1.07 (0.65-1.76) |  | 0.98 (0.70-1.35) | 0.94 (0.65-1.38) | 1.05 (0.60-1.82) |
| Q3 | 1.04 (0.70-1.55) | 1.17 (0.68-2.04) | 1.21 (0.75-1.95) |  | 1.05 (0.76-1.45) | 1.07 (0.74-1.55) | 1.07 (0.61-1.87) |
| Q4 | 1.07 (0.72-1.61) | 1.42 (0.83-2.45) | 0.82 (0.48-1.40) |  | 1.08 (0.78-1.49) | 0.83 (0.56-1.24) | 1.72 (1.04-2.85) |
| Q5 | 1.26 (0.85-1.86) | 1.40 (0.81-2.41) | 1.05 (0.63-1.73) |  | 1.12 (0.81-1.55) | 0.99 (0.67-1.45) | 1.37 (0.80-2.36) |
| P_trend_ ^*^ | 0.35 | 0.27 | 0.81 |  | 0.40 | 0.47 | 0.05 |
| Per SD increase | 1.02 (0.89-1.16) | 1.01 (0.85-1.21) | 1.05 (0.91-1.22) |  | 0.99 (0.89-1.11) | 0.95 (0.84-1.08) | 1.10 (0.93-1.31) |
| **Body fat %** |  |  |  |  |  |  |  |
| Q1 | 1.00 | 1.00 | 1.00 |  | 1.00 | 1.00 | 1.00 |
| Q2 | 1.08 (0.72-1.63) | 1.19 (0.69-2.07) | 1.03 (0.61-1.74) |  | 0.89 (0.64-1.23) | 1.01 (0.69-1.47) | 0.74 (0.43-1.28) |
| Q3 | 1.24 (0.83-1.85) | 1.35 (0.79-2.33) | 1.39 (0.84-2.28) |  | 1.01 (0.73-1.39) | 0.96 (0.66-1.42) | 1.16 (0.70-1.91) |
| Q4 | 1.11 (0.73-1.68) | 1.38 (0.79-2.38) | 0.94 (0.55-1.63) |  | 0.93 (0.67-1.30) | 0.88 (0.59-1.31) | 0.98 (0.57-1.67) |
| Q5 | 1.36 (0.91-2.02) | 1.33 (0.76-2.31) | 1.24 (0.74-2.06) |  | 1.07 (0.77-1.47) | 1.01 (0.68-1.50) | 1.21 (0.72-2.03) |
| P_trend_ ^*^ | 0.16 | 0.27 | 0.54 |  | 0.65 | 0.80 | 0.29 |
| Per SD increase | 1.09 (0.96-1.24) | 1.12 (0.94-1.34) | 1.01 (0.86-1.19) |  | 0.98 (0.88-1.09) | 0.93 (0.82-1.05) | 1.08 (0.91-1.29) |
| **Whole body fat mass to whole body fat free mass** |  |  |  |  |  |  |  |
| Q1 | 1.00 | 1.00 | 1.00 |  | 1.00 | 1.00 | 1.00 |
| Q2 | 1.06 (0.70-1.59) | 1.16 (0.67-2.02) | 1.05 (0.62-1.78) |  | 0.88 (0.64-1.22) | 1.00 (0.69-1.46) | 0.73 (0.42-1.27) |
| Q3 | 1.18 (0.79-1.77) | 1.27 (0.74-2.19) | 1.40 (0.85-2.31) |  | 1.00 (0.72-1.37) | 0.94 (0.64-1.39) | 1.15 (0.70-1.90) |
| Q4 | 1.05 (0.69-1.60) | 1.31 (0.75-2.28) | 0.93 (0.54-1.62) |  | 0.91 (0.65-1.27) | 0.87 (0.58-1.30) | 0.95 (0.55-1.63) |
| Q5 | 1.32 (0.89-1.96) | 1.31 (0.76-2.27) | 1.27 (0.76-2.11) |  | 1.06 (0.76-1.46) | 1.00 (0.68-1.48) | 1.19 (0.71-1.99) |
| P_trend_ ^*^ | 0.21 | 0.30 | 0.52 |  | 0.69 | 0.77 | 0.32 |
| Per SD increase | 1.03 (0.91-1.16) | 1.02 (0.86-1.21) | 1.06 (0.92-1.22) |  | 0.99 (0.89-1.10) | 0.94 (0.83-1.06) | 1.09 (0.92-1.30) |
| **BMI** |  |  |  |  |  |  |  |
| Q1 | 1.00 | 1.00 | 1.00 |  | 1.00 | 1.00 | 1.00 |
| Q2 | 1.17 (0.81-1.69) | 0.72 (0.43-1.20) | 1.75 (1.09-2.81) |  | 1.04 (0.75-1.43) | 1.01 (0.69-1.48) | 1.20 (0.70-2.08) |
| Q3 | 1.26 (0.84-1.88) | 1.38 (0.85-2.24) | 1.16 (0.66-2.06) |  | 1.01 (0.73-1.40) | 0.98 (0.67-1.44) | 1.43 (0.84-2.42) |
| Q4 | 0.84 (0.57-1.25) | 0.57 (0.33-0.99) | 1.21 (0.73-1.99) |  | 1.31 (0.96-1.79) | 1.16 (0.80-1.69) | 1.64 (0.97-2.76) |
| Q5 | 1.01 (0.67-1.52) | 1.01 (0.61-1.67) | 0.89 (0.50-1.59) |  | 1.03 (0.74-1.44) | 0.93 (0.63-1.39) | 1.31 (0.75-2.26) |
| P_trend_ ^*^ | 0.44 | 0.73 | 0.26 |  | 0.40 | 0.97 | 0.16 |
| Per SD increase | 0.95 (0.83-1.07) | 0.98 (0.83-1.16) | 0.91 (0.78-1.06) |  | 1.03 (0.92-1.14) | 0.98 (0.87-1.12) | 1.10 (0.93-1.31) |
|  |  |  |  |  |  |  |  |
| **TFMI (kg/m^2^)** |  |  |  |  |  |  |  |
| Q1 | 1.00 | 1.00 | 1.00 |  | 1.00 | 1.00 | 1.00 |
| Q2 | 1.12 (0.75-1.66) | 1.30 (0.76-2.23) | 1.12 (0.67-1.86) |  | 0.93 (0.69-1.28) | 0.98 (0.68-1.42) | 0.86 (0.50-1.47) |
| Q3 | 0.99 (0.66-1.50) | 1.01 (0.57-1.79) | 1.27 (0.77-2.09) |  | 1.01 (0.73-1.39) | 0.91 (0.62-1.33) | 1.25 (0.76-2.07) |
| Q4 | 1.15 (0.77-1.73) | 1.47 (0.85-2.52) | 0.98 (0.57-1.69) |  | 0.89 (0.64-1.24) | 0.91 (0.62-1.34) | 1.00 (0.58-1.72) |
| Q5 | 1.28 (0.86-1.90) | 1.36 (0.78-2.35) | 1.12 (0.67-1.88) |  | 1.14 (0.83-1.57) | 0.99 (0.67-1.46) | 1.37 (0.82-2.27) |
| P_trend_ ^*^ | 0.24 | 0.26 | 0.88 |  | 0.92 | 0.82 | 0.18 |
| Per SD increase | 1.05 (0.93-1.20) | 1.09 (0.92-1.30) | 0.99 (0.84-1.16) |  | 0.99 (0.89-1.10) | 0.94 (0.83-1.07) | 1.10 (0.93-1.30) |
| **Trunk fat %** |  |  |  |  |  |  |  |
| Q1 | 1.00 | 1.00 | 1.00 |  | 1.00 | 1.00 | 1.00 |
| Q2 | 0.96 (0.64-1.44) | 0.95 (0.54-1.66) | 1.21 (0.74-1.99) |  | 0.89 (0.64-1.22) | 0.90 (0.62-1.30) | 0.92 (0.53-1.55) |
| Q3 | 1.03 (0.69-1.53) | 1.24 (0.73-2.10) | 1.03 (0.61-1.73) |  | 1.03 (0.75-1.43) | 0.96 (0.65-1.41) | 1.22 (0.72-2.05) |
| Q4 | 1.12 (0.75-1.67) | 1.32 (0.78-2.25) | 1.11 (0.66-1.85) |  | 0.93 (0.67-1.31) | 0.90 (0.61-1.34) | 1.07 (0.61-1.85) |
| Q5 | 1.30(0.88-1.92) | 1.34 (0.79-2.30) | 1.17 (0.70-1.95) |  | 1.05 (0.75-1..48) | 0.91 (0.60-1.37) | 1.34 (0.78-2.31) |
| P_trend_ ^*^ | 0.13 | 0.14 | 0.73 |  | 0.68 | 0.69 | 0.23 |
| Per SD increase | 1.07 (0.94-1.22) | 1.11 (0.93-1.32) | 1.01 (0.86-1.19) |  | 0.96 (0.86-1.08) | 0.92 (0.80-1.04) | 1.07 (0.89-1.28) |
| **Trunk fat to trunk fat free mass** |  |  |  |  |  |  |  |
| Q1 | 1.00 | 1.00 | 1.00 |  | 1.00 | 1.00 | 1.00 |
| Q2 | 0.97 (0.64-1.46) | 1.04 (0.59-1.82) | 1.12 (0.67-1.85) |  | 0.84 (0.61-1.15) | 0.87 (0.60-1.26) | 0.80 (0.46-1.39) |
| Q3 | 1.01 (0.67-1.51) | 1.20 (0.70-2.06) | 1.03 (0.61-1.72) |  | 1.01 (0.73-1.39) | 0.92 (0.63-1.35) | 1.25 (0.75-2.08) |
| Q4 | 1.15 (0.78-1.71) | 1.35 (0.79-2.31) | 1.16 (0.70-1.92) |  | 0.89 (0.64-1.25) | 0.85 (0.57-1.27) | 1.05 (0.61-1.82) |
| Q5 | 1.31 (0.88-1.93) | 1.40 (0.82-2.41) | 1.13 (0.67-1.88) |  | 1.06 (0.76-1.48) | 0.92 (0.61-1.38) | 1.32 (0.77-2.27) |
| P_trend_ ^*^ | 0.11 | 0.13 | 0.64 |  | 0.63 | 0.69 | 0.18 |
| Per SD increase | 1.08 (0.95-1.22) | 1.11 (0.93-1.31) | 1.01 (0.86-1.19) |  | 0.97 (0.87-1.09) | 0.92 (0.81-1.05) | 1.08 (0.91-1.28) |
| **Leg fat mass (kg)** |  |  |  |  |  |  |  |
| Q1 | 1.00 | 1.00 | 1.00 |  | 1.00 | 1.00 | 1.00 |
| Q2 | 1.02 (0.71-1.48) | 1.20 (0.73-1.98) | 0.85 (0.49-1.46) |  | 0.82 (0.59-1.13) | 0.95 (0.66-1.38) | 0.51 (0.26-1.01) |
| Q3 | 1.14 (0.79-1.65) | 1.18 (0.70-1.98) | 1.10 (0.65-1.86) |  | 1.02 (0.75-1.39) | 0.99 (0.69-1.44) | 1.08 (0.62-1.89) |
| Q4 | 0.78 (0.51-1.21) | 1.03 (0.59-1.82) | 0.54 (0.27-1.07) |  | 0.94 (0.68-1.31) | 0.89 (0.60-1.32) | 1.07 (0.60-1.92) |
| Q5 | 0.97 (0.67-1.43) | 1.16 (0.69-1.94) | 0.80 (0.45-1.41) |  | 1.22 (0.87-1.70) | 1.14 (0.76-1.70) | 1.40 (0.78-2.54) |
| P_trend_ ^*^ | 0.59 | 0.78 | 0.26 |  | 0.23 | 0.76 | 0.09 |
| Per SD increase | 0.98 (0.86-1.11) | 1.01 (0.86-1.20) | 0.93 (0.76-1.13) |  | 1.02 (0.91-1.14) | 0.97 (0.85-1.10) | 1.15 (0.94-1.41) |
| **Ratio of trunk fat mass to leg fat mass** |  |  |  |  |  |  |  |
| Q1 | 1.00 | 1.00 | 1.00 |  | 1.00 | 1.00 | 1.00 |
| Q2 | 1.09 (0.72-1.64) | 1.40 (0.81-2.45) | 1.01 (0.60-1.70) |  | 1.09 (0.72-1.64) | 0.84 (0.57-1.25) | 0.78 (0.46-1.34) |
| Q3 | 0.79 (0.52-1.22) | 1.40 (0.79-2.47) | 1.37 (0.83-2.26) |  | 0.79 (0.52-1.22) | 1.24 (0.85-1.80) | 1.07 (0.64-1.81) |
| Q4 | 1.40 (0.94-2.06) | 1.40 (0.80-2.47) | 1.15 (0.69-1.93) |  | 1.40 (0.94-2.06) | 0.87 (0.57-1.33) | 0.82 (0.46-1.45) |
| Q5 | 1.47 (0.97-2.22) | 1.58 (0.90-2.79) | 1.04 (0.61-1.78) |  | 1.47 (0.97-2.22) | 0.83 (0.51-1.34) | 1.39 (0.82-2.34) |
| P_trend_ ^*^ | 0.02 | 0.17 | 0.73 |  | 0.68 | 0.59 | 0.20 |
| Per SD increase | 1.10 (0.96-1.25) | 1.12 (0.94-1.34) | 1.00 (0.85-1.18) |  | 0.98 (0.88-1.10) | 0.89 (0.76-1.04) | 1.10 (0.92-1.31) |
| **WC (cm)** |  |  |  |  |  |  |  |
| Q1 | 1.00 | 1.00 | 1.00 |  | 1.00 | 1.00 | 1.00 |
| Q2 | 1.10 (0.73-1.65) | 1.41 (0.80-2.46) | 0.92 (0.55-1.53) |  | 0.96 (0.71-1.29) | 0.91 (0.64-1.31) | 1.24 (0.76-2.01) |
| Q3 | 1.29 (0.89-1.89) | 0.99 (0.55-1.78) | 0.98 (0.61-1.59) |  | 1.09 (0.80-1.50) | 1.04 (0.71-1.51) | 1.19 (0.70-2.01) |
| Q4 | 1.30 (0.89-1.90) | 1.38 (0.79-2.42) | 1.19 (0.75-1.88) |  | 1.15 (0.83-1.61) | 1.12 (0.75-1.67) | 1.49 (0.87-2.54) |
| Q5 | 1.45 (0.96-2.18) | 1.79 (1.02-3.15) | 1.11 (0.66-1.87) |  | 0.96 (0.69-1.33) | 0.95 (0.64-1.40) | 1.03 (0.59-1.79) |
| P_trend_^*^ | 0.05 | 0.08 | 0.44 |  | 0.81 | 0.85 | 0.69 |
| Per SD increase | 1.12 (0.98-1.27) | 1.14 (1.05-1.36) | 1.07 (0.90-1.27) |  | 1.04 (0.94-1.16) | 1.02 (0.90-1.16) | 1.09 (0.92-1.29) |
| **WHR** |  |  |  |  |  |  |  |
| Q1 | 1.00 | 1.00 | 1.00 |  | 1.00 | 1.00 | 1.00 |
| Q2 | 0.86 (0.57-1.30) | 0.98 (0.56-1.71) | 0.86 (0.52-1.43) |  | 0.99 (0.70-1.38) | 1.08 (0.72-1.60) | 0.80 (0.45-1.42) |
| Q3 | 1.27 (0.87-1.86) | 1.30 (0.77-2.19) | 1.03 (0.64-1.67) |  | 1.26 (0.92-1.74) | 1.20 (0.81-1.77) | 1.46 (0.89-2.40) |
| Q4 | 1.20 (0.80-1.80) | 1.50 (0.88-2.54) | 0.98 (0.59-1.64) |  | 1.14 (0.83-1.59) | 1.15 (0.78-1.70) | 1.32 (0.79-2.19) |
| Q5 | 1.42 (0.96-2.09) | 1.50 (0.88-2.55) | 1.23 (0.76-2.00) |  | 1.08 (0.77-1.51) | 1.08 (0.72-1.61) | 0.98 (0.56-1.70) |
| P_trend_^*^ | 0.03 | 0.04 | 0.33 |  | 0.43 | 0.64 | 0.47 |
| Per SD increase | 1.12 (0.99-1.26) | 1.15 (0.98-1.34) | 1.06 (0.91-1.24) |  | 1.04 (0.93-1.15) | 1.03 (0.91-1.16) | 1.06 (0.90-1.25) |

- All models were adjusted for age at enrollment, education, physical activity, alcohol intake, smoking, height, red meat intake, processed meat intake, fruits and vegetable intake, folate supplement intake, Vitamin D supplement intake, history of diabetes. CI= confidence interval; HR= hazard ratio; SD= standard deviation; FMI= fat mass index; TFMI= trunk fat mass index; WC= waist circumference; WHR= waist to hip ratio
- ^*^All tests were two-sided.
- Ranges:
- Men: FMI: ≤3.7, 3.8-4.5, 4.6-5.1, 5.2-5.7, >5.7; body fat %: ≤16.4, 16.5-19.2, 19.3-21.4, 21.5-23.7, >23.7; whole body fat to whole body fat free mass: ≤0.20, 0.21-0.24, 0.25-0.27, 0.28-0.31, >0.31; BMI: ≤22.1, 22.2-23.3, 23.4-23.8, 23.9-24.5, >24.5; TFMI: ≤2.22; 2.23-2.81, 2.82-3.25, 3.25-3.68, >3.68; trunk fat %: ≤17.2, 17.3-20.9, 21.0-23.7, 23.8-26.6, >26.6; trunk fat mass to trunk fat free mass: ≤0.20, 0.21-0.26, 0.27-0.31, 0.31-0.36, >0.36; leg fat mass: ≤3.3, 3.4-3.8, 3.9-4.2, 4.3-4.6, >4.6; ratio of trunk fat mass to leg fat mass: ≤1.93, 1.94-2.21, 2.22-2.44, 2.45-2.69, >2.69; WC: ≤82, 83-85, 86-88, 89-92, >92; WHR: ≤0.85, 0.86-0.88, 0.89-0.91, 0.92-0.94, >0.94 for quintiles 1,2 3, 4, and 5, respectively
- Women: FMI: ≤6.0, 6.1-6.9, 7.0-7.6, 7.7-8.4, >8.4; body fat %: ≤27.4, 27.5-30.5, 30.6-32.8, 32.9-35.1, >35.1; whole body fat to whole body fat free mass: ≤0.38, 0.39-0.44, 0.45-0.49, 0.50-0.54,> 0.54; BMI: ≤21.4, 21.5-22.6, 22.7-23.5, 23.6-24.2, >24.2; TFMI: ≤2.86; 2.87-3.44, 3.45-3.90, 3.91-4.39, >4.39; trunk fat %: ≤23.8, 23.9-27.8, 27.9-30.6, 30.7-33.6, >33.6; trunk fat mass to trunk fat free mass: ≤0.31, 0.32-0.38, 0.39-0.44, 0.45-0.50, >0.50; leg fat mass: ≤6.8, 6.9-7.6, 7.7-8.2, 8.3-8.8, >8.8; ratio of trunk fat mass to leg fat mass: ≤1.06, 1.07-1.19, 1.20-1.30, 1.31-1.42, >1.43; WC: ≤70, 71-74, 75-77, 78-80.3, >80.3; WHR: ≤0.74, 0.75-0.77, 0.78-0.80, 0.81-0.83, >0.83 for quintiles 1,2 3, 4, and 5, respectively
